# The Competition of Termination and Shielding to Evaluate the Success of Surface-Initiated Reversible Deactivation Radical Polymerization

**DOI:** 10.3390/polym12061409

**Published:** 2020-06-23

**Authors:** Francisco J. Arraez, Paul H. M. Van Steenberge, Dagmar R. D’hooge

**Affiliations:** 1 Laboratory for Chemical Technology, Department of Materials, Textiles and Chemical Engineering, Ghent University, Technologiepark 125, Zwijnaarde, 9052 Ghent, Belgium; franciscojose.arraezhernandez@ugent.be (F.J.A.); paul.vansteenberge@ugent.be (P.H.M.V.S.); 2 Centre for Textile Science and Engineering, Department of Materials, Textiles and Chemical Engineering, Ghent University, Technologiepark 70A, Zwijnaarde, 9052 Ghent, Belgium

**Keywords:** stochastic modeling, surfaces, conformations, kinetics, apparent livingness

## Abstract

One of the challenges for brush synthesis for advanced bioinspired applications using surface-initiated reversible deactivation radical polymerization (SI-RDRP) is the understanding of the relevance of confinement on the reaction probabilities and specifically the role of termination reactions. The present work puts forward a new matrix-based kinetic Monte Carlo platform with an implicit reaction scheme capable of evaluating the growth pattern of individual free and tethered chains in three-dimensional format during SI-RDRP. For illustration purposes, emphasis is on normal SI-atom transfer radical polymerization, introducing concepts such as the apparent livingness and the molecular height distribution (MHD). The former is determined based on the combination of the disturbing impact of termination (related to conventional livingness) and shielding of deactivated species (additional correction due to hindrance), and the latter allows structure-property relationships to be identified, starting at the molecular level in view of future brush characterization. It is shown that under well-defined SI-RDRP conditions the contribution of (shorter) hindered dormant chains is relevant and more pronounced for higher average initiator coverages, despite the fraction of dead chains being less. A dominance of surface-solution termination is also put forward, considering two extreme diffusion modes, i.e., translational and segmental. With the translational mode termination is largely suppressed and the living limit is mimicked, whereas with the segmental mode termination occurs more and the termination front moves upward alongside the polymer layer growth. In any case, bimodalities are established for the tethered chains both on the level of the chain length distribution and the MHD.

## 1. Introduction

The bio-functionalization of solid substrates by means of polymer layers is relevant for the modification of their physicochemical properties and to control the response of these substrates with respect to external or environmental stimuli such as temperature, pH and ionic strength of the medium in which these substrates are immersed [1,2,3,4]. Hence, the synthesis of well-defined polymer structures constitutes the molecular basis for the development of advanced functional surfaces, which find applications in areas such as sample purification, biosensing platforms, antifouling coatings, platforms for controlled cell culture as well as drug and gene delivery [5,6,7,8,9,10]. The application range of surfaces functionalized with polymer depends to a great extent on the conformation of the polymer chains that make up the polymeric layer [11,12,13]. The conformation adopted by surface-tethered polymer chains is governed by a competition between the high entropic cost for these chains to stretch out to their maximum length and the excluded volume interactions between their segments. This competition is to a first approximation influenced by the average chain grafting density (σ_chains_), which is linked to the average initiator coverage, and more in detail by the spectrum of values of the radius of gyration (*R_g,surf_*) of the individual polymer chains [14,15,16].

Conventionally, two main or average regimes of three-dimensional (3D) conformations have been introduced to describe the polymer layer on a surface, i.e., the so-called mushroom and brush regime [17,18,19]. Recent work of Arraez et al. [15] focusing on surface initiated living polymerization (SIP) with chain initiation and propagation at the surface and in the solution highlighted that a classification based on a single average can be biased. The polymer layer cannot be described solely by introducing an average conformation but should be at least described by percentages of chains belonging to the two aforementioned conformation regimes, also considering a third intermediate regime termed brush-like to highlight a mixed contribution of the two main regimes [15,20]. Based on literature data [14,21] and dedicated analysis considering complete simulated *R_g,surf_* distributions, these authors proposed to classify each chain according to:

(1) $$\mathrm{mushroom}\;\mathrm{conformation}\;R_{g,surf}^{2}<\frac{1}{{4\sigma }_{\mathrm{chains}}}$$

(2) $$\mathrm{brush}\text{-}\mathrm{like}\;\mathrm{conformation}\;\frac{1}{{4\sigma }_{\mathrm{chains}}}\leq {\;R}_{g,surf}^{2}\leq \;10\times \left(\frac{1}{{4\sigma }_{\mathrm{chains}}}\right)$$

(3) $$\mathrm{brush}\;\mathrm{conformation}\;R_{g,surf}^{2}\;>\;10\times \left(\frac{1}{{4\sigma }_{\mathrm{chains}}}\right)$$

It should be stressed that these three equations only reflect a heuristic approach to compare several synthesis routes with respect to their success in achieving a desired conformation. In other words, the scaling factors of 1/4 and 10/4, as used to differentiate between the conformation of individual tethered chains, cannot be claimed as universal. They are only employed for a guide-of-the-eye and a fast assessment of the brush quality at the molecular level.

In the present work, the usefulness of Equations (1)–(3) is further explored for surface-initiated reversible-deactivation radical polymerization (SI-RDRP) as this synthesis technique is frequently employed to develop novel functional materials [22,23]. In this technique, RDRP initiator molecules are chemically anchored on the surface (*R*_0*,surf*_*X* species) and by only moderately increasing the temperature (e.g., to 343 K) and subtle control of the chemical structure of the reactants and the initial concentrations, tethered chains with well-defined molar mass and chain composition, low dispersity and controlled architecture and conformation result [24,25,26]. Furthermore, the values for σ_chains_ and the thickness of the polymer layers prepared using SI-RDRP are usually higher than those obtained using other synthesis strategies [14,25,27].

Three variants of SI-RDRP have attracted significant attention for the preparation of polymer layers on the surface: (i) surface-initiated atom transfer radical polymerization (SI-ATRP) [28,29,30,31,32,33], (ii) surface-initiated stable free radical polymerization (SI-SFRP) [34,35,36] and (iii) surface-initiated reversible-addition fragmentation chain transfer (SI-RAFT) polymerization [37,38,39,40,41]. Molecular control is each time achieved by a dynamic equilibrium (exchange) between active radicals and dormant (macro)species that allows for slow but quasi-simultaneous growth of tethered and solution chains. This is done while keeping a predominant concentration of dormant species and a low contribution of dead polymer chains, thus a minimization of termination on a chain basis (e.g., 10%).

**Figure 1:**
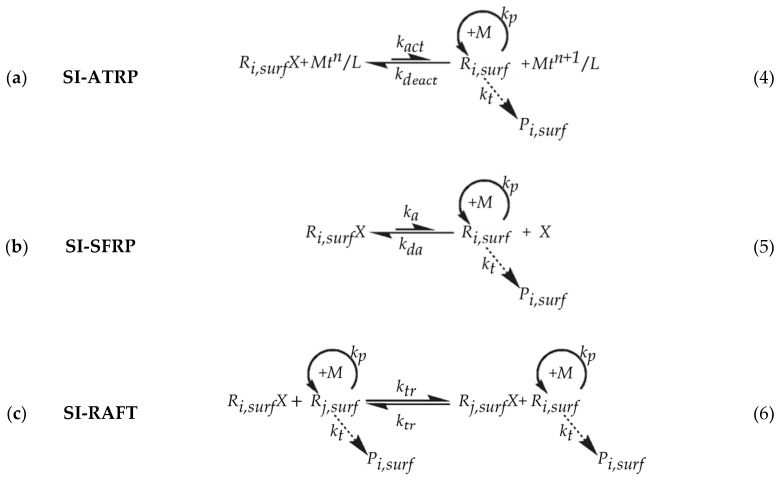
Principle of (**a**) surface-initiated atom transfer radical polymerization (SI-ATRP; Equation (4)) [28,29,30,31,32,33], (**b**) surface-initiated stable free radical polymerization (SI-SFRP; Equation (5)) [34,35,36] and (**c**) surface-initiated reversible-addition fragmentation chain transfer (SI-RAFT; Equation (6)) polymerization [37,38,39,40,41], depicting for simplicity only reactions on the surface (“surf”). In parallel, similar reactions can occur in the solution or between surface and solution species. In (**c**) the degenerative mechanism is depicted for simplicity while the initial radical source is not explicitly represented; surface-tethered active macroradicals with chain length *i* and *j* are represented as *R_i,surf_* and *R_j,surf_*, while surface-tethered dormant species are denoted as *R_i,surf_X* and *R_j,surf_X*. *P_i,surf_* stands for a surface-tethered dead chain with chain length *i*, and *M* represents monomer. Specific details (**a**) SI-ATRP: transition metal compound (*M_t_^n^*/*L*; activator; *M_t_^n^*: transition metal with oxidation state *n*; *L*: ligand) and higher oxidation state metal complex (*M_t_^n^*^+1^/*L*; deactivator); (**b**) SI-SFRP: stable free radical (X; typically nitroxide radical); *k_a/da_*: activation/deactivation rate coefficient; *k_tr_*: exchange “rate coefficient”; *k_p/t_*: propagation/termination rate coefficient.

As shown in Figure 1, which only focuses on reactions with surface species for simplicity, the main difference between the three variants lies in the method of radical generation. In SI-ATRP (Equation (4)), surface-tethered dormant species (*R_i,surf_X*; *i*: chain length) can undergo a reversible redox process catalyzed by a transition metal compound (*M_t_^n^*/*L*; *Mt^n^*: transition metal with oxidation state *n*; *L*: ligand) that leads to the generation of an active surface-tethered macroradical (*R_i,surf_*; *i*: chain length) and a higher oxidation state metal complex (*M_t_^n^*^+1^/*L*), which serves as a deactivator. This radical takes up a limited number of monomer units and is then rapidly deactivated again, awaiting a new activation-growth-deactivation cycle at a later stage. SI-SFRP (Equation (5)) parallels SI-ATRP but utilizes stable (persistent) radicals (*X*; typically nitroxide radicals) as deactivator. Unlike the previous two processes, SI-RAFT (Equation (6)) polymerization controls chain growth through a fast reversible exchange (transfer “reaction”) among surface-tethered macroradicals and surface-tethered dormant species using an efficient RAFT chain transfer agent (CTA). The initial radical source is a conventional radical initiator, which is strictly not needed for the other two variants. In all cases, in the solution, similar reactions can take place (removal of “surf” in Figure 1) and termination can also occur in a crossing way thus involving one solution and one surface species. These extra reactions complicate the overall kinetic description with rate coefficients possessing different dimensions, including s^−1^, dm^2^ mol^−1^ s^−1^ and L mol^−1^ s^−1^.

**Figure 2:**
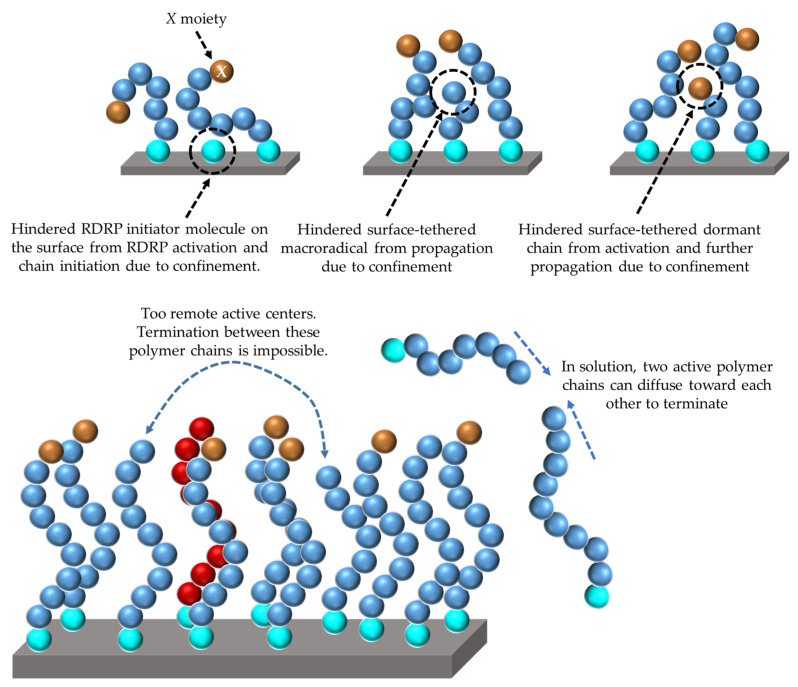
Visualization of confinement in surface initiation—reversible deactivation radical polymerization (SI-RDRP), leading to different reaction probabilities for reactions on the surface and in the solution. For illustration purposes, emphasis is on chain initiation, propagation, activation/deactivation at the top and termination at the bottom. Here, RDRP initiator molecules are represented as cyan spheres, monomer units either in dormant or active polymer chains are represented as blue spheres, and monomer units in dead polymer chains are represented as red spheres. In addition, the “X” chain-ends of dormant polymer chains are depicted as brown spheres to differentiate themselves from the active species.

Several challenges still exist regarding the design of SI-RDRP with specifically the correct quantification of the end-group functionality (EGF), i.e., the fraction of chains being dormant and thus not dead. With less availability of *X* moieties, the RDRP mechanism becomes less active and thus a disturbance of the desired growth pattern is realized. To control macroscopic properties (e.g., surface wettability, adhesive properties, and lubrication) and to ensure optimal functioning of the functionalized substrate, a detailed characterization and design of molecular properties such as EGF, chemical composition and conformation is needed. However, the characterization of the molecular properties of surface-tethered chains is usually a burdensome task that generally requires the chains to be cleaved from the surface. In addition, the determination of σ_chains_ is usually more onerous than measuring the (average) molar mass [14,42], therefore making it more difficult to access detailed experimental information on the conformations of the surface-tethered polymer chains. In contrast, experimental analysis of the chains in solution is relatively straightforward but not recommended [15,42,43,44], as the kinetics for chain formation in the solution and on the surface are completely different, due to a very strong impact of confinement effects exerted by the presence of the surface. For example, the likelihood that two active chains at the surface can terminate with each other is much lower than if those two chains are free in the solution, as conceptually illustrated in Figure 2 (bottom). Furthermore, other confinement effects are highlighted in this figure at the top, selecting shielding for chain initiation, propagation and RDRP activation/deactivation as examples. If no empty space is available, radicals and dormant chains can become apparently inactive. They still possess the correct functional moiety, but the other reactant or the potential product formed cannot escape the polymer layer anymore. Currently, it is unclear what the effect of shielding is along the RDRP process, although Arraez et al. [15] recently theoretically demonstrated that shielding on chain initiation and propagation highly impact the SIP kinetics, specifically for high average initiator coverages.

Interestingly, computer simulations represent a powerful strategy to deal with the limitations that arise from the experimental characterization of polymer layers [12,14,23,43,45,46] and to test certain confinement hypotheses. For example, several researchers [47,48] used the deterministic method of moments, which aims only at modeling averages, to introduce a so-called migration or hopping/rolling mechanism to understand time dependencies of termination reactions in SI-RDRP. Here, a sufficiently fast RDRP activation/deactivation (in the brush regime) could alter the local radical concentrations to increase termination rates counteracting the confinement effect. Most stochastic models are in turn based on simplified constant reaction probabilities but allow an explicit positioning of the monomer molecules/units in lattice format, with important contributions based on the 3D bond fluctuation model (BFM) and the dynamic lattice liquid model (DLL) [42,44,49,50,51,52,53,54,55,56]. Arraez et al. [15] very recently developed so-called matrix-based kinetic Monte Carlo (*k*MC) simulations in which BFM is used to represent monomer (unit) positions in 3D but obeying for the first time synthesis time dependent reaction probabilities, as determined by explicitly tracking concentration variations for chains on the surface and in the solution region near that surface. The *k*MC platform, as applied for SIP with only chain initiation and propagation on the surface and in the solution, allows a reliable and unique prediction of molecular average and distributed properties of both tethered and free chains. This includes the calculation of the temporal evolution of average chain lengths and the chain length distribution (CLD), as well as σ_chains_ and the 3D visualization of the conformation of the individual chains within the polymer layer. Hence, it is worthwhile further exploring the potential of the principles behind the matrix-based *k*MC platform developed for the more complex case of RDRP.

In the present work, we therefore move forward by extending our *k*MC platform for brush synthesis from SIP to a general RDRP, hence, acknowledging the occurrence of termination reactions on the one hand and activation/deactivation reactions on the other hand. This is done in a lumped manner correcting the associated rate coefficients with, for simplicity, a single equilibrium coefficient for the activation/deactivation process (*K_eq_*), formally considering a normal ATRP mechanism. For termination particularly, an additional confinement correction is performed based on the available 3D space to let a chain (or part of it) move in the polymer layer. Limiting cases mimicking translational and segmental diffusion are considered, further underlining the novelty of the present work to capture in detail the effect of SI-RDRP reaction conditions on chain growth control and EGF preservation. It is demonstrated that an apparent livingness is required with the translational mode, as the dominant contributor for disturbance of the SI-RDRP mechanism is a shielding effect for reactions involving dormant species and not the actual formation of dead tethered chains, at least for the selected *K_eq_* value. In contrast, termination reactions significantly occur for the segmental mode. Here, chain length dependencies are relevant, which along the RDRP termination dominantly occur at larger layer heights to form a quite heterogeneous height profile. In this context, a molecular height distribution (MHD) is introduced, which together with the variation of the (average) monomer coverage as a function of height, enables brushes to be quantified in a more macroscopic thus physical way.

## 2. Kinetic Modeling Methodology

The current work builds on our previous *k*MC contribution [15] focused on SIP, in which termination reactions are by definition not implemented and only chain initiation and propagation reactions are allowed either on a flat surface or in the solution near the surface. In particular, our previous work revealed that the confinement kinetic effects experienced by the surface-tethered polymer chains lead to an increased formation of hindered shorter (living) chains that are unable to further propagate (cf., Figure 2; top middle). At relatively long synthesis times, the shielding effect induces a bimodal CLD for surface-tethered polymer chains compared to the unimodal CLD for free polymer chains which are represented on average by longer polymer chains. More importantly, a conformational characterization of the simulated surface-tethered polymer chains in terms of the mushroom, brush-like and brush conformation regimes (Equations (1)–(3)) was proposed through the generation of the distributions of the radius of gyration from the explicit visualization of the polymer chains on the surface.

**Figure 3:**
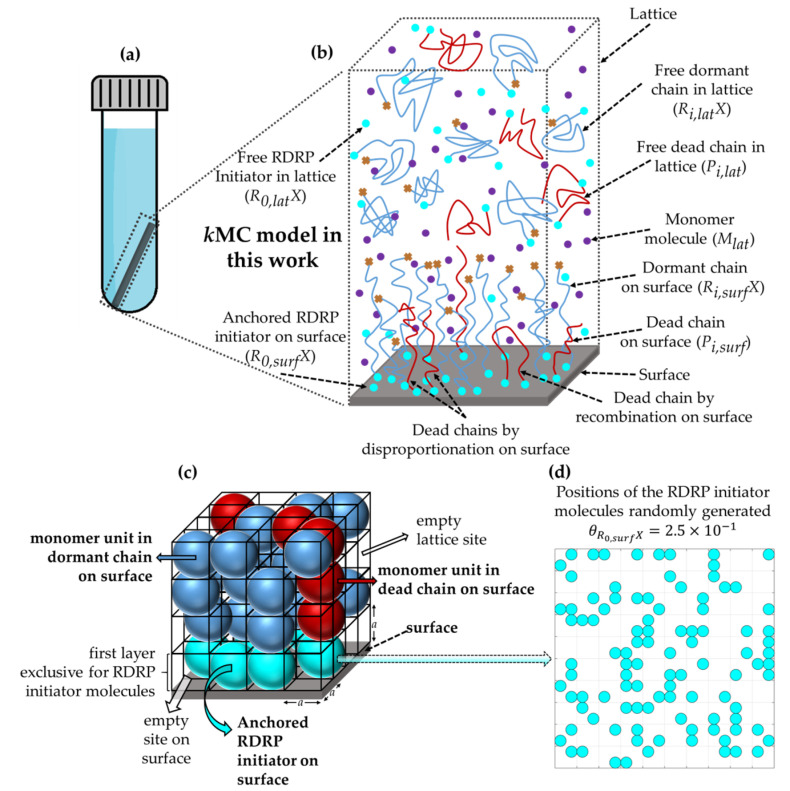
Principles of kinetic Monte Carlo (kMC) model developed in the present work for surface-initiated reversible deactivation radical polymerization (SI-RDRP), starting from the principles in Arraez et al. [15] addressing surface-initiated living polymerization (SIP). As shown in (**a**), emphasis is on a flat surface, and as shown in (**b**), this model covers the kinetics of reactions taking place both on the surface (surf) as well as in the vicinity of the surface (lat). This is the region delimited by the dotted gray rectangle in (**a**) including the substrate, with blue lines corresponding either to surface-tethered dormant chains (*R_i,surf_X* species; *i*: chain length; depicted for simplicity as highly stretched chains) or free dormant chains (*R_i,lat_X* species; depicted as coiled chains), red lines to dead polymer chains that reside as tethered chains on the surface (*P_i,surf_* species) or free in the solution (*P_i,lat_* species), cyan circles being RDRP initiator molecules in both phases (*R*_0*,surf*_*X* and *R*_0*,lat*_*X* species) and purple circles the (free) monomer molecules near the surface (*M_lat_* species). As shown in (**c**), in this model chemical species are represented as spheres within cubes from a cubic lattice with a lattice constant *a*. Here, chemically anchored RDRP initiator molecules on the surface are represented in cyan, while monomer units in the surface-tethered dormant chains and the dead chain on the surface are represented in blue and red, respectively. In (**d**) the associated 2D visualization of the random positioning of RDRP initiator molecules chemically anchored on a flat surface but obeying a given average RDRP initiator coverage ($\theta_{R_{0,sur}X}$ = 2.5 × 10^−1^) is shown. Upon removing the dead species, the original subfigures as introduced in Arraez et al. [15] are obtained.

In the present section, the main aspects of this previous work are briefly highlighted and specific emphasis is on the novel implementation steps to grasp the confinement effect of termination and activation/deactivation on the SI-RDRP kinetics. For illustration purposes the RDRP mechanism is assumed to be the normal ATRP one (cf. Figure 1; top). Focus is on the tackling of SI-ATRP reactions that take place in the near-surface region and on the actual surface, as shown in Figure 3a by enclosing the region by the dotted-gray rectangle, including the full gray surface. A representation of all the chemical species considered to participate in these chemical reactions is covered in Figure 3b. The actually implemented lumped reactions are summarized in Table 1. Lumped descriptions are needed as otherwise too large a computational cost results in the framework of an explicit visualization of the 3D conformational variations of chains as a function of synthesis time. In what follows, it is first explained how the chemical species are represented in 3D format and then it is elaborated how the lumped reactions need to be interpreted to allow for a representative stochastic sampling of the reactions involved. Here, apparent lumped rate coefficients appropriately rescaled with *K*_eq_ are utilized, making the *k*MC model an implicit one.

### 2.1. Lattice Framework and Representation of Solution and Anchored Chemical Species

A mean-field approximation (MFA) is applied for the representation of the free chemical species in the vicinity of the surface (species with subscript “*lat*”), and the 3D positions of these chemical species are not explicitly tracked. Conversely, the 3D positions of the chemically anchored initiator molecules and the monomer units of the dormant and dead chains on the surface are explicitly tracked and stored within a 3D cubic lattice, based on a modified implementation of Shaffer’s version [57] of the bond fluctuation model (BFM), as shown in Figure 3c. As explained further upon cross-termination (thus between a solution and surface species), the lattice is also used to validate if sufficient space is available to execute the reaction. Similarly, for reactions involving monomer it is verified that the extra monomer unit can be placed.

The cubic lattice is built on a three-dimensional Cartesian coordinate system with a lattice constant *a* = 1 that corresponds to an actual length of 0.78 nm, which is representative for molecules with a molar mass of about 100 g·mol^−1^, as explained in previous work [15]. Periodic boundary conditions are applied in the *x* and *y* direction, while both limits of *z* direction are modeled as impenetrable substrates with the flat surface located in the horizontal *x-y* plane at *z* = 0. In addition, as can be seen in Figure 3c, anchored molecules or monomer units are represented as spheres within a unit cell of the cubic lattice. The center of each sphere coincides with the geometric center of the unit cell where it is contained. Chemically anchored RDRP initiator molecules on the surface (*R*_0*,surf*_*X*) are represented as cyan spheres, while monomer units in both the dormant chains (*R_i,surf_X*) and the dead chains on the surface (*P_i,surf_*) are represented in blue and red, respectively.

The first layer of the lattice (0 ≤ *z* ≤ 1) is exclusive to *R*_0,*surf*_*X* molecules, whereas monomer units of dormant or dead chains on the surface can occupy the empty lattice sites only from the second layer upward (*z* > 1). Bond vectors ($\vec{b}$) within the lattice are defined in terms of the distance between two molecule centers. The allowed bond vectors are represented by all possible permutations and sign inversion of the following set of vectors: $\vec{b}\in P(1,0,0)\cup P(1,1,0)\cup P(1,1,1)$ [57]. Thus, the resulting (scaled) bond lengths formally allowed in the model are 1, $\sqrt{2}$,$\;\sqrt{3}$. The excluded volume condition, in which one part of a polymer chain cannot occupy space that is already occupied by another part of the same or another polymer chain, is enforced by forbidding double occupancy of the primary unit cell for the involved monomer units. In addition, chain crossing within the lattice is not allowed, and this is done by tracking the position of the midpoints of all bond vectors for all monomer units and prohibiting them from overlapping.

### 2.2. Matrix-Based Kinetic Monte Carlo Algorithm Implementation

#### 2.2.1. Random Selection of Overall Reactions before Possible Confinement Correction

The matrix-based *k*MC algorithm to follow SI-RDRP kinetics on and near the surface is based on previous work [61,62,63,64,65,66] for the design of living and RDRP processes, starting from the stochastic rules pioneered by Gillespie [67]. Reaction events and the time at which these events occur are randomly selected according to the reaction probabilities calculated from so-called MC reaction rates expressed in s^−1^:

(7) $$\tau =\frac{1}{a_{0}}\ln\left(\frac{1}{r_{1}}\right)=\frac{1}{\sum a_{j,k}}\ln\left(\frac{1}{r_{1}}\right)$$

(8) $$\sum_{j=1}^{\mu }a_{j,k}\geq \;r_{2}a_{0}$$

in which $a_{0}$ stands for the total MC reaction rate, which is calculated from the sum of the individual Monte Carlo reaction rates following from the so-called MC reaction channels ($a_{0}=\sum a_{j,k}$). Furthermore, $r_{1}$ and $r_{2}$ are uniformly distributed random numbers in the interval (0, 1], and μ is the smallest integer that satisfies Equation (8).

As shown in Table 1, the reaction possibilities (MC channels) correspond to those of chain initiation, propagation, termination by recombination and termination by disproportionation. A distinction is also made between two phases depending on whether the active species resides on the surface (subscript *k* = *surf*) or is free in the solution near the surface (subscript *k* = *lat*). Formally, the reactions in Table 1 do not stand for single (elementary) reactions, but instead they are described by a series of activation, the actual core reaction and the potential deactivation. In case radicals are explicitly tracked, the number of molecules (and 3D positions) goes up tremendously, as in well-conducted SI-RDRP processes termination reactions are rare events. It is therefore better to formally calculate the reaction kinetics based on the dormant species and adapt the corresponding “rate coefficients”. Hence, Table 1 displays lumped chemical reactions, and the corresponding reaction equations are expressed based on the lumped apparent rate coefficients ($k_{j,k}^{\mathrm{app}}$ values).

The determination of $k_{j,k}^{\mathrm{app}}$ (dm^n^ L^−1^ s^−1^; *n* = 2, 3) depends on the reaction type and is done based on the associated elementary rate coefficient ($k_{j,k}$; dm^n^ L^−1^ s^−1^; *n* = 2, 3) and the equilibrium coefficient ($K_{\mathrm{eq}})$, always taking the same value regardless of the phase for simplicity. For instance, for propagation on the surface, it suffices to focus on the total (ATRP) concentration of surface macroradicals ($\left[R_{\mathrm{surf}}\right]$), which can be roughly assessed by [68]:

(9) $$\left[R_{\mathrm{surf}}\right]\;\approx {\;K}_{\mathrm{eq}}{\left[R_{,\mathrm{surf}}X\right]}_{0}\frac{{\left[{\mathrm{Mt}}^{n}/L\right]}_{0}}{{\left[{\mathrm{Mt}}^{n+1}/L\right]}_{0}}$$

This implies for the (surface overall) polymerization rate:

(10) $$R_{p}^{surf}{=k}_{p,\mathrm{lat}\text{-}\mathrm{surf}}\left[M_{\mathrm{lat}}\right]\left[R_{\mathrm{surf}}\right]\sim {\;K}_{\mathrm{eq}}k_{p,\mathrm{lat}\text{-}\mathrm{surf}}\left[M_{\mathrm{lat}}\right]$$

explaining that in Table 1 it is stated that

(11) $$k_{p,\mathrm{lat}\text{-}\mathrm{surf}}^{\mathrm{app}}=K_{\mathrm{eq}}k_{p,\mathrm{lat}\text{-}\mathrm{surf}}$$

formally capturing all scaling in *K_eq_* making this also an apparent (but constant) parameter. Following the same procedure, the lumped apparent rate coefficient for propagation in the solution near the surface ($k_{p,\mathrm{lat}}^{\mathrm{app}}$) as well as the lumped apparent rate coefficients for chain initiation in both phases ($k_{i,\mathrm{lat}\text{-}\mathrm{surf}}^{\mathrm{app}}\;$and $k_{i,\mathrm{lat}\;}^{\mathrm{app}}$) are determined. Similarly, for the (overall) termination rate, it can be written down to a first approximation that [69]

(12) $$R_{t}^{surf}{=k}_{t}{\left[R_{\mathrm{surf}}\right]}^{2}\sim {\left(K_{eq}\right)}^{2}k_{t,\mathrm{surf}}$$

explaining that in Table 1 it is stated that

(13) $$k_{t,\mathrm{surf}}^{\mathrm{app}}={\left(K_{eq}\right)}^{2}k_{t,\mathrm{surf}}$$

It should be noted that the $k_{j,k}^{\mathrm{app}}$ values in Table 1 are ball-park values that are just scaled in a proper way without claiming that the individual values are all correct. In other words, they are just selected considering Equations (11) and (13), which at least allows the correct order of the related reaction probabilities to be reflected. Future work should be devoted to their more detailed (even time-dependent) determination but this is outside the scope of the present work. Here, we perform implicit *k*MC simulations for SI-RDRP aiming at a general understanding of kinetic differences between the solution region near the surface and the actual surface. A quite controlled SI-RDRP is selected, thus with a fair but limited share of dead chain formation.

As previously mentioned (Equations (7) and (8)), the determination of the most probable reaction path and the time at which the selected reaction takes place is based on the determination of the MC reaction rates in s^−1^, which in turn needs the calculation of MC rate coefficients ($k_{j,k}^{MC}$) in s^−1^. Here, the $k_{j,k}^{\mathrm{MC}}$ values are determined from the corresponding $k_{j,k}^{\mathrm{app}}$ values and are based on the molecularity of the associated reaction, i.e., unimolecular or bimolecular. Furthermore, depending on whether the reaction takes place only on the surface (“*surf*”) or in the solution (“*lat*”) or between the solution and the surface (“*lat-surf*”), an appropriate scaling is needed, considering either a suitable control surface area ($S_{MC}$) or control solution volume ($V_{MC}$). In this work, only bimolecular reactions are considered (Table 1) and the determination of the MC rate coefficients is carried out using Equations (14), (15) and (16) in which *N_A_* the Avogadro number:

(14) $$k_{j,\mathrm{surf}}^{\mathrm{MC}}=\frac{k_{j,\mathrm{surf}}^{\mathrm{app}}}{S_{MC}N_{A}}$$

(15) $$k_{j,\mathrm{lat}\text{-}\mathrm{surf}}^{\mathrm{MC}}=\frac{k_{j,\mathrm{lat}\text{-}\mathrm{surf}}^{\mathrm{app}}}{V_{\mathrm{MC}}N_{A}}\;$$

(16) $$k_{j,\mathrm{lat}}^{\mathrm{MC}}=\frac{k_{j,\mathrm{lat}}^{\mathrm{app}}}{V_{\mathrm{MC}}N_{A}}$$

#### 2.2.2. Initialization and Possible Correction for Confinement

Under so-called reference conditions, the initial number of initiator molecules either chemically anchored on the surface ($N_{R_{0,\mathrm{surf}}X}$) or free in the lattice ($N_{R_{0,\mathrm{lat}}X}$) is chosen to be equal to 1 × 10^5^ molecules, while the initial number of monomer molecules ($N_{M_{\mathrm{lat},0}}$) is set to be 2 × 10^7^ molecules, which corresponds to a target chain length (TCL) of 100 in both phases. $R_{0,\mathrm{surf}}X$ molecules are placed on the surface by generating a priori uniformly distributed random positions ($\vec{r}\equiv (r_{x},r_{y},r_{z})$) of the molecules’ centers in the *x-y* plane and setting $r_{z}$ = 0.5. Figure 3d shows an example of a 2D projection of the random positioning of RDRP initiator molecules chemically anchored on a reduced but representative portion of the flat surface for a given RDRP initiator reference surface coverage ($\theta_{R_{0,\mathrm{sur}}X}$). In this work, the reference conditions are chosen to represent a surface area of approximately 2.4 × 10^5^ nm^2^ with $\theta_{R_{0,\mathrm{sur}}X}$ = 2.5 × 10^−1^. In addition, the volume of the vicinity of the surface is approximately equal to 1.7 × 10^7^ nm^3^.

As previously stated, the 3D positions of monomer molecules ($M_{\mathrm{lat}})$ and free RDRP initiator molecules ($R_{0,\mathrm{lat}}X)$ are not explicitly tracked within the lattice, and a MFA is applied throughout the lattice, which is acceptable as these species are very small and thus mobile. Accordingly, their overall concentrations are automatically updated each time a reaction involving these molecules is carried out. The execution of selected chemical reactions based on overall MC rates ($a_{j,k}$ values) without the participation of species tethered to the surface in the solution is carried out in the traditional way based on previous work [61,62,63,64,65], as no further considerations regarding confinement matter. However, if a reaction concerning chemical species on the surface is chosen, a confinement correction is needed. Here a distinction is made between reactions with one “radical” (chain initiation and propagation) and two “radicals” (termination).

If a chain initiation or propagation reaction taking place on the surface is selected (reaction 1 and 3 in Table 1), then the availability of space around the corresponding reactive centers is evaluated based on the allowed bond lengths. In this context, each empty lattice site around the reactive center is allowed to act as a possible free monomer spot for the corresponding reaction to proceed. More in detail, for both reactions, first a $R_{0,\mathrm{surf}}X$ molecule or $R_{i,\mathrm{surf}}X$ polymer chain is randomly selected from a pool of available molecules through a random number *r*_3_ in the interval (0, 1]. For the chain initiation reaction, the first element of the new polymer chain is positioned just on top of the chosen $R_{0,\mathrm{surf}}X$ molecule, provided that the lattice position is initially empty. As for the propagation reaction, all the neighboring sites at the reactive chain-end of the $R_{i,\mathrm{surf}}X$ chain are examined to determine whether these are empty (thus available) or occupied by another monomer unit of the same or another polymer chain, based on the permutation of the set of vectors $\vec{b}$. If the top position of the selected $R_{0,\mathrm{surf}}X$ molecule or all neighboring sites around the reactive center in $R_{i,\mathrm{surf}}X$ are occupied, then the reactions are considered to fail and the corresponding chemical species are removed from the calculation of the overall MC reaction rates ($a_{i,\mathrm{surf}}$ and $a_{p,\mathrm{surf}}$). As a consequence, $R_{0,\mathrm{surf}}X$ or $R_{i,\mathrm{surf}}X$ species involved in the failed reaction event are labeled as hindered from chain initiation and further propagation, respectively. On the other hand, if one or more sites are available upon attempting a propagation reaction on the surface, then the site that will undergo the reaction, that is, where the new monomer unit will be located, is selected with uniform probability by using a new random number *r_4_* in the interval (0, 1]. Note that the chains on the surface are fixated but the kinetics are correctly accounted for. The time scale of the kinetics is the one that matters in the present work, rather than the smaller time scale for the local reconfiguration, consistent with literature data [15,54].

With respect to the termination reactions either by recombination or by disproportionation, the execution in the *k*MC model depends on whether the (lumped) reaction occurs between two $R_{i/j,\mathrm{surf}}X$ species or if the (lumped) termination is taking place between a $R_{i,\mathrm{surf}}X$ species and a $R_{j,\mathrm{lat}}X$ species. Regarding the (lumped) termination between two surface species, the reaction is executed only if the two chain-ends are next to each other, based on the allowed bond lengths (1, √2, √3), and the chains are not hindered from propagation. Preliminary simulations (with the selected *K*_eq_) in the present work revealed that such a situation is very unlikely, implying that if two chains become active on the surface it is very unlikely they are close enough. As for the (lumped) mixed termination between $R_{i,\mathrm{surf}}X$ and $R_{j,\mathrm{lat}}X$, the reaction is executed by first selecting the two polymer chains with the given chain lengths *i* and *j* in both phases, based on two random numbers *r*_5_ and *r*_6_ in the interval (0, 1], and then looking for the feasibility to place the free polymer chain “on top” of its surface counterpart. Here, two modes of spatial allocation are proposed, resembling limiting cases of translational and segmental diffusion. These two diffusion modes are therefore referred to as the “segmental mode” and the “translational mode”. The latter mode is considered as the reference in the *k*MC model.

In the segmental mode, the selected free polymer chain $R_{j,\mathrm{lat}}X$ is provisionally constructed by randomly placing one by one its monomer units in available lattice sites by using random numbers in the interval (0, 1] and based on the allowed bond lengths 1, √2, √3, starting at the reactive center of the tethered chain. If upon selecting a new position this position is occupied, then the *k*MC algorithm selects a new position randomly and tries to still position the corresponding monomer unit. If all positions are occupied, it means that the free chain cannot be positioned and the reaction event fails. Furthermore, backtracking to a previous monomer unit is not allowed to correct the growth path. The transitional mode is similar in implementation but is much more stringent regarding mobility as it mimics a single-shot termination attempt. This implies that if during the assigning of the positions of the individual monomer units of the free chain a selected lattice site is occupied, the *k*MC algorithm directly stops the provisional chain construction and the termination reaction event fails immediately. A common feature between the two termination modes comes forward if a recombination reaction event is successfully attempted. Then, the provisional positions of the free chain combine with those of the chain tethered to the surface, resulting in a new polymer dead chain on the surface $P_{i+j,\mathrm{surf}}$.

## 3. Results and Discussion

Unless stated otherwise, an SI-RDRP kinetic study is conducted under reference conditions as described in Section 2.2.2 (thus $\theta_{R_{0,\mathrm{sur}}X}$ = 2.5 × 10^−1^), the kinetics parameters in Table 1 and the stringent transitional mode for the termination reactions between the surface and free solution polymer chains. A more conventional approach is first followed with specific focus on the evaluation of the EGF evolution in both phases. Confinement effects are then put forward based on a so-called apparent livingness and a molecular height distribution (MHD). This relevance of distributions is further highlighted, ultimately extending the 3D conformational study on SIP [15] to fully characterize the different populations of RDRP-made polymer chains residing on the flat surface. The effect of surface overcrowding is investigated as well, for which the three optimal $\theta_{R_{0,\mathrm{sur}}X}$ values (1.0 × 10^−1^, 2.5 × 10^−1^ and 5.0 × 10^−1^) in agreement with experimental contributions [70] and our previous work [15] are adopted. Finally, the two allocation modes, i.e., the segmental mode and the translational mode for diffusion, are compared for a SI-RDRP otherwise under reference conditions.

### 3.1. From Conventional Molecular Properties to Molecular Height Distribution and Apparent Livingness

**Figure 4:**
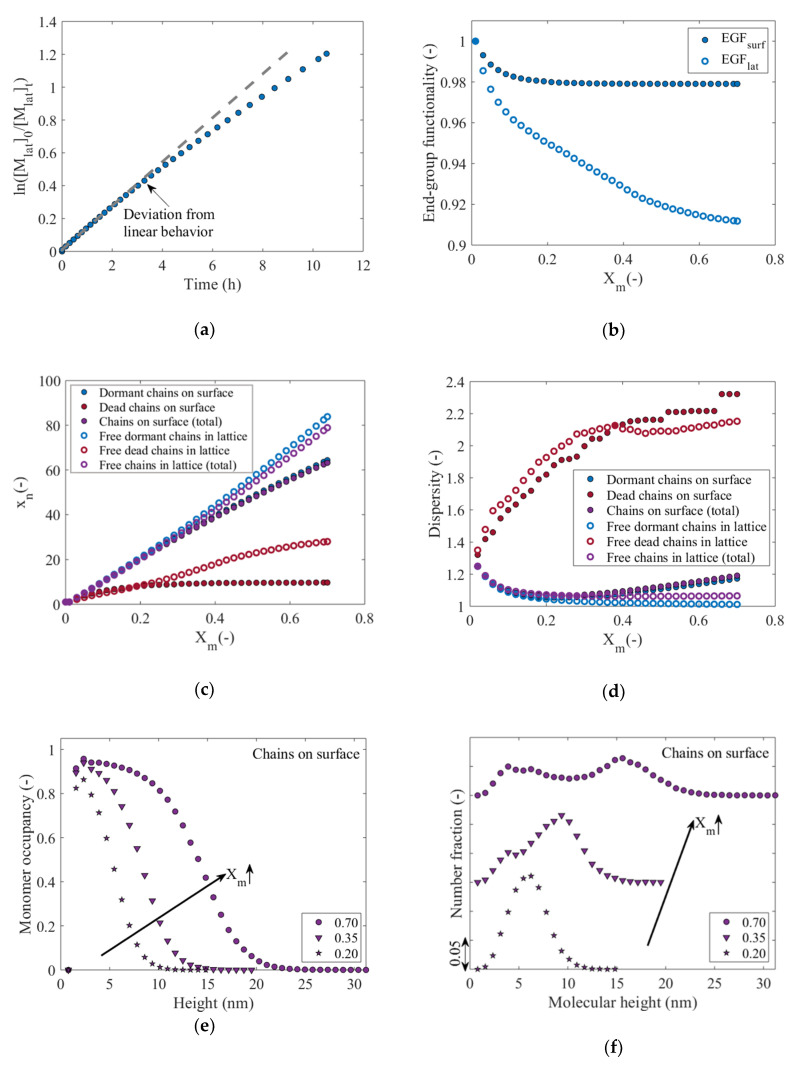
(**a**) Evolution of ${\ln([M}_{\mathrm{lat}}{]}_{0}/{{[M}_{\mathrm{lat}}]}_{t})$ vs time under reference surface-initiated reversible deactivation radical polymerization (SI-RDRP) conditions (translational mode). Corresponding (**b**) end-group functionality (EGF) on surface (black-contoured full blue circles; *EGF_surf_*) and free dormant chains in solution near the surface (blue-contoured empty circles; *EGF_lat_*) as a function of monomer conversion (*X_m_*). (**c**) Number average chain length (*x_n_*) and (**d**) dispersity for dormant (blue symbols) and dead chains (red symbols) as well as the total values (purple) on surface (full symbols) or in solution (empty symbols) as a function of *X_m_*. (**e**) Monomer occupancy along the lattice height (z direction) in nm at 3 *X_m_* values and (**f**) molecular height distribution (MHD) at three *X_m_* values. Simulation conditions: amount of RDRP initiator molecules $N_{R_{0,\mathrm{lat}}X}{=N}_{R_{0,\mathrm{surf}}X}$ = 1 × 10^5^, target chain length (TCL) of 100 in both phases (initial number of monomer molecules $N_{M}$ = 2 × 10^7^) and average surface coverage $\theta_{R_{0,\mathrm{sur}}X}$ = 2.5 × 10^−1^; kinetic parameters in Table 1.

The simulated ${\ln([M}_{\mathrm{lat}}{]}_{0}/{{[M}_{\mathrm{lat}}]}_{t})$ profile (black-contoured full blue circles) up to a monomer conversion *X_m_* = 0.7 under reference SI-RDRP conditions is shown in Figure 4a. For comparison, a dashed gray line has been included to highlight the downward curvature deviation of the simulated profile from the linear increase representative of first-order kinetics for a perfectly living RDRP process with instantaneous initiation. This behavior has also been observed for SIP and attributed to confinement thus the difference in the kinetics on and near the surface [15]. Figure 4b displays the EGF values as a function of *X_m_* to show that *EGF_surf_* (black-contoured full blue circles) rapidly decreases down to a value around 0.98 for a monomer conversion *X_m_* = 0.1, after which it remains approximately constant. On the other hand, *EGF_lat_* (blue-contoured empty circles) gradually decreases as a function of monomer conversion, achieving a value of just below 0.92 at *X_m_* = 0.7. This difference reveals that termination reactions, which lead to a decrease in EGF, are more prone to take place in the solution, wherein chains can more easily diffuse toward each other, while they are more restricted to take place on the surface, as previously covered in Figure 2. For completeness, the evolution of the reaction probabilities as a function of monomer conversion, as well as the absolute variation of the chemical species in the simulated process as a function of monomer conversion *X_m_* are included in Figure S1 in Supporting Information.

Figure 4c,d show the evolution of the number-average chain length (*x_n_*) and dispersity of the different populations of polymer chains (dormant/dead; blue/ed symbols) as well as the associated result for the total population (data represented in purple) on the surface (full symbols) and in the near-surface solution (empty symbols) as a function of *X_m_*. From Figure 4c it is observed that for low *X_m_* (*X_m_* ≤ 0.2), *x_n_* values evolve in a similar way per polymer population. However, for higher *X_m_* (*X_m_* > 0.2), a marked deviation is observed in both phases. It can be seen that *x_n_* of the free dormant polymer chains increases to a value slightly above 80 at *X_m_* = 0.7, whereas *x_n_* of the surface-tethered dormant polymer chains increases only to a value of around 60. The variation of the total *x_n_* follows a very similar trend to that of the dormant polymer chains, since the latter represent most of the chains that make up the total, as shown in Figure 4b.

Likewise, it is observed in Figure 4c that the free dead chains reach higher *x_n_* values than their counterpart on the surface, although in both cases the maximum values are well below the values shown for the total, indicating that termination reactions take place mainly between shorter radicals and especially at low *X_m_*, which is typical for a well-controlled ATRP process. Figure 4d shows that the dispersity of the free dormant polymer chains displays a decreasing trend toward a value very close to one at *X_m_* = 0.7, reflecting a good molecular control in the solution as expected from a well-conducted RDRP. On the other hand, the dispersity values of the dormant surface-tethered polymer chains steadily increase up to ca. 1.2 at *X_m_* = 0.7. The dispersity of dead chains in both phases climbs up even more strongly to values between 2.2 and 2.4 at *X_m_* = 0.7, although with little or no contribution to the total dispersity, due to the low proportion of dead chains in the polymerization system (again Figure 4b). The results in Figure 4a–d show that the reactions on the surface and in the lattice take place in different chemical environments, and consequently, the molecular properties for both phases differ considerably. This confirms that the widespread strategy in describing the molecular properties of the surface-tethered polymer chains based on the properties of the free polymer chains in solution [30,31,71,72] leads to a biased representation of the properties of the polymer layer, consistent with previous simulation studies [15,42,44,53].

Furthermore, an important physical characteristic of the polymer layer is the (average) monomer occupancy profile, which is defined as the ratio of the number of monomer units in the surface-tethered polymer chains (either dormant or dead) to the total number of sites available per parallel *x-y* plane to the surface. Figure 4e shows the monomer occupancy profiles at three *X_m_* values as a function of the lattice height in nm. It follows that these profiles shift to the right as *X_m_* increases, indicating growth in the height of the polymer layer on the surface. A broadening is also observed, implicitly highlighting shielding effects. More in detail, Figure 4f shows the associated MHDs of the surface-tethered polymer chains, which are constructed based on the highest points of individual polymer chains that make up the polymer layer. Unlike the monomer occupancy profiles which are obtained from a collective contribution of all the polymer chains on the surface at a given height, the MHD reflects the individual character of each polymer chain within the polymer layer from a top view. The MHDs in Figure 4f exhibit a transition from a distribution with monomodal character at *X_m_* = 0.2 to one with bimodal character at *X_m_* = 0.7, further indicating a heterogeneous layer growth with possibly strong shielding effects.

**Figure 5:**
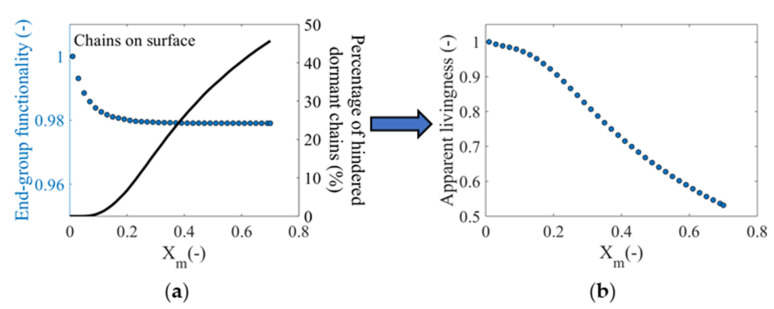
(**a**) Evolution of the end-group functionality of surface-tethered dormant chains as well as the percentage of dormant chains that become hindered from RDRP activation (-growth-deactivation) within the polymer layer due to shielding as a function of monomer conversion (*X_m_*). (**b**) By combining both characteristics from (**a**), it is possible to determine the evolution of a new molecular property, the apparent livingness of the polymer layer. This property refers to the actual fraction of surface-tethered dormant chains within the polymer layer that can participate in additional propagation events. Results are based on the same (reference) reaction conditions (translational mode) as in Figure 4 and the kinetic parameters in Table 1.

This increased heterogeneity of the polymer layer can be explained based on Figure 5a, which shows the *X_m_* dependence of the percentage of hindered dormant chains on the surface incapable of further growth by activation-propagation-deactivation cycles, due to lack of space around the X chain-ends as a consequence of confinement on the surface (solid black line). It follows that the SI-RDRP can be described by firstly showing an induction period at very low *X_m_* (*X_m_* < 0.1) in which polymer chains on the surface are unhindered from net propagation. According to Arraez et al. [15], this period is characterized by incomplete chain initiation on the surface that leads to a sparsely populated surface in which the excluded volume interactions are negligible. However, at *X_m_* ≥ 0.1 the percentage of hindered dormant polymer chains shows a progressive increase toward a value just below 50%. In this context, the confinement effect on the surface leads to an accumulation of apparently inactive dormant polymer chains of short chain lengths, compared to the fraction of dormant tethered chains that can continue to propagate upon activation-growth-deactivation with a further increase of their corresponding chain lengths. This behavior logically exerts a strong influence on the MHD, leading to the bimodality observed in Figure 4f.

In Figure 5a, the EGF_surf_ variation is also repeated alongside the aforementioned fraction of hindered dormant chains. After combining these two characteristics, a new property is introduced in Figure 5b denoted as the apparent livingness of the polymer layer. The apparent livingness refers to the actual fraction of surface-tethered polymer chains that can continue to grow through the RDRP mechanism and can be further modified. A distinctive attribute of the modified solid substrates for biological applications through the growth of a polymer layer on the substrate surface is the feasibility of the extensibility or subsequent functionalization of polymer chains within the polymer layer. For this, the polymerization process must be designed paying attention to the synthesis conditions to guarantee at first sight a high *EGF_surf_*, which is linked to the availability of surface-tethered polymer chains that can be modified in subsequent stages to the primary polymerization. However, as shown in Figure 5a, a description of the extensibility or further chemical modification of the polymer layer based solely on *EGF_surf_* is incorrect. For the reference conditions, approximately half of the chains with the desired end-group functionality are trapped within the polymer layer, therefore preventing access to and modification of these polymer chains. In this way, it is clear that for a complete characterization of the polymer layer on the surface, the determination of *EGF_surf_* and the percentage of hindered dormant polymer chains on the surface is required. As can be seen in Figure 5b, the apparent livingness of the polymer layer decreases as a function of *X_m_*, showing that only 50% of the (dormant) polymer chains on the surface can continue to grow within the polymer layer at *X_m_* = 0.7.

### 3.2. From Molecular Distributions to a 3D Vizualization of Tethered Polymer Chains

**Figure 6:**
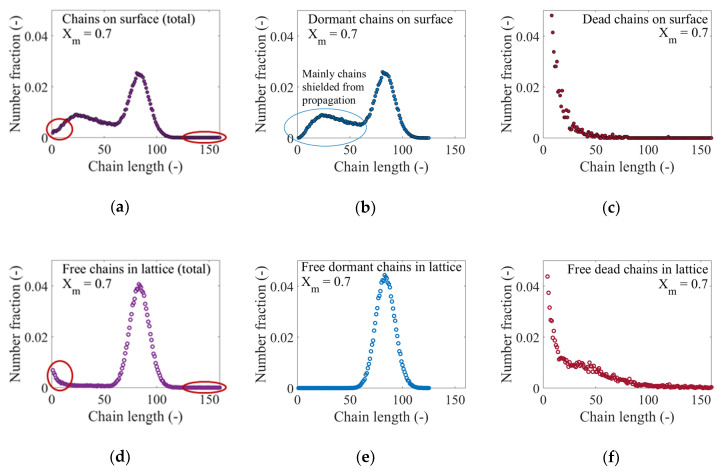
Top: Number chain length distribution of (**a**) all surface-tethered polymer chains (black-contoured full purple circles), (**b**) surface-tethered dormant chains (black-contoured full blue circles) and (**c**) surface-tethered dead polymer chains (black-contoured full red circles). Bottom: Number chain length distribution of (**d**) all free polymer chains (purple-contoured empty circles), (**e**) free dormant chains (blue-contoured empty circles) and (**f**) free dead polymer chains (red-contoured empty circles). In (**a**,**d**) the regions circled in red represent the contributions of dead polymer chains to the CLD of the total polymer species in both phases. These distributions are obtained at a monomer conversion *X_m_* = 0.7. Same (reference) conditions (translational mode) as in Figure 4 and the kinetic parameters in Table 1. The evolution of these distributions as a function of monomer conversion is included in Figure S2 in Supporting Information.

The increasing contribution of hindered dormant tethered polymer chains in Figure 5a leads to bimodal CLDs for the surface-tethered dormant polymer chains, as shown in Figure 6a,b, which depict CLDs of the dormant and all polymer chains on the surface at *X_m_* = 0.7. In both cases, two main peaks are identified. The first peak centers around a chain length of just over 20 and extends from a chain length of 1 to approximately 60, and mainly comprises polymer chains that are hindered in the polymer layer. The second narrower peak, which is centered around a chain length around 80, is mainly made up of polymer chains with the ability to further increase their chain length through activation-propagation-deactivation reactions. Differences between both distributions exist, with the development of a longer tail for the total one as well as a subtle increase in the fraction of polymer chains of very short chain lengths (<10). These differences can be explained by the contribution of the surface-tethered dead polymer chains to the total. As can be seen in Figure 6c, the CLD of dead chains on the surface is characterized by a pseudo-exponential distribution with the largest fraction of polymer chains having a chain length just below 10 (dead chains formed at low *X_m_*) and a longer tail that extends to a chain length up to 160 (dead chains formed at higher *X_m_*). In the interest of comparison, Figure 6d‒f show the CLD of the total population of free polymer chains in the near-surface solution as well as the CLD of the free dormant polymer chains and free dead chains, at *X_m_* = 0.7. As expected, for a solution RDRP, narrow unimodal distributions result for the total population of free polymer chains and free dormant chains in the solution, whose peaks center around a chain length slightly greater than 80, reflecting again good molecular control. The solution dead polymer CLD (Figure 6f) is similar in shape but has a higher impact on the total one, as witnessed by a stronger increase at the lower chain length region in Figure 6d.

**Figure 7:**
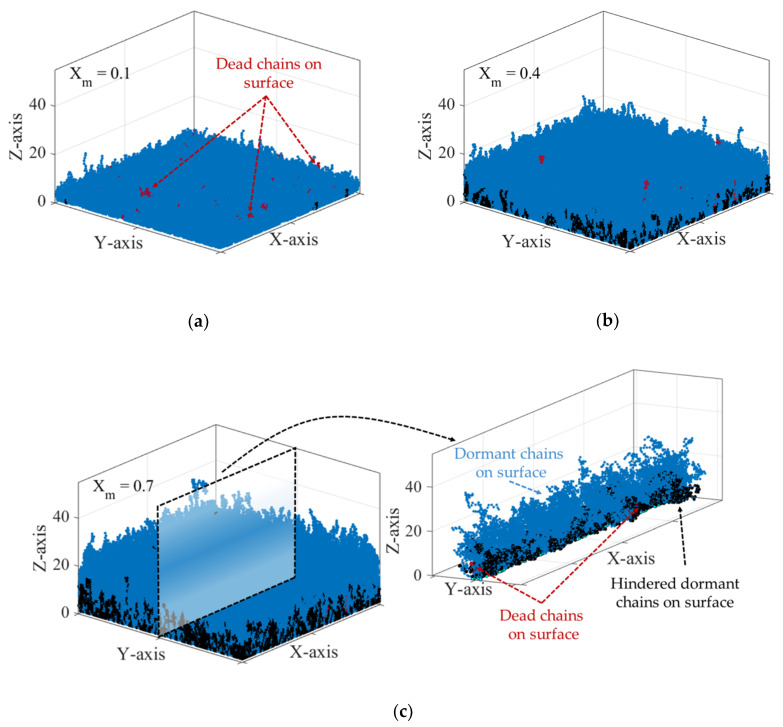
Snapshot of the polymer layer grown; same references conditions as in Figure 4, Figure 5 and Figure 6 thus with specifically the translational mode to describe the termination between a solution and surface active species. The snapshots are taken at three different monomer conversions: (**a**) *X_m_* = 0.1; (**b**) *X_m_* = 0.4; (**c**) *X_m_* = 0.7. Surface-tethered dormant chains are represented in blue, dead chains on the surface are represented in red, while the hindered dormant chains that cannot continue to propagate by means of the RDPR activation-growth-deactivation mechanismdue to shielding are represented in black. A zoom of the middle section (*Y*-axis) of the polymer layer in (**c**) at *X_m_* = 0.7 to facilitate the differentiation of these three groups of polymer chains.

Three snapshots of the polymer layer obtained at *X_m_* equal to 0.1, 0.4 and 0.7 are shown in Figure 7a–c. In these representations, the surface-tethered dormant polymer chains are depicted as blue chains, dead chains on the surface are colored red, while hindered dormant chains are represented as black chains. The presence of dead polymer chains—reminding the stringent translational mode—is notable at low *X_m_* as shown in Figure 7a, while it is practically impossible to spot these chains within the polymer layer at high *X_m_* (Figure 7c). This agrees with the previous results shown in Figure 4b and Figure 6a in which it is established that the formation of dead chains takes place mostly at low *X_m_*, leading to dead chains with short chain length that reside at the bottom of the growing polymer layer. The increasing trend of the percentage of hindered dormant chains, as previously shown in Figure 5a, is also visualized in these snapshots by an increase in the proportion of black chains in the representations of the polymer layer. In addition, a snapshot of the polymer chains grown in the middle section of the surface (*y*-axis) is included in Figure 7c, where it can be appreciated more elegantly how the hindered polymer chains, as well as the dead chains on the surface, are located in the innermost areas of the polymer layer, whereas the reactive centers of the dormant chains that still have the ability to propagate by activation/propagation/deactivation cycles are located more towards the upper end of the polymer layer.

**Figure 8:**
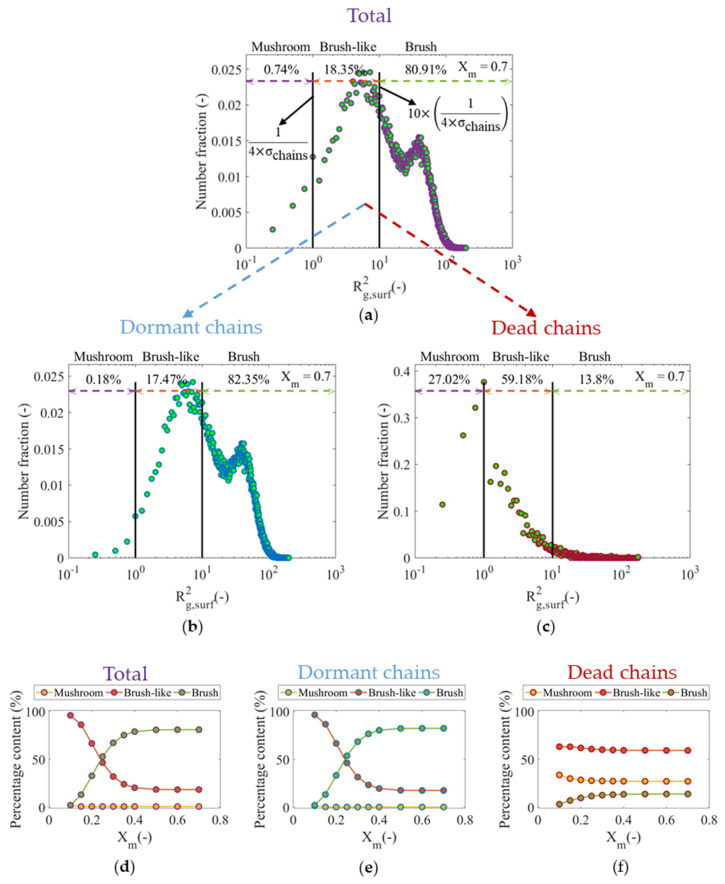
(**a**) Distribution of the squared radius of gyration ($R_{g,\mathrm{surf}}^{2}$) of all individual surface-tethered polymer chains (purple-contoured full green circles) grown on the surface at a monomer conversion $X_{m}$ = 0.7. (**b**) Corresponding distribution for surface-tethered dormant chains (blue-contoured full green circles) and (**c**) dead polymer chains on surface (red-contoured full green circles). The two, black guide-of-the-eye vertical lines are calculated from Equations (1) and (3). Also shown are the associated percentages for mushroom, brush-like and brush chains; (**d**–**f**) these percentages are now given as a function of $X_{m}$, differentiating between all chains (total), dormant and dead surface-tethered chains. Conditions as in Figure 4, Figure 5, Figure 6 and Figure 7 (translational mode) and kinetic parameters in Table 1.

This explicit representation of the spatial distribution of the polymer chains on the surface allows for the determination of the conformational properties of the individual polymer chains such as the squared end-to-end distance ($R_{e,\mathrm{surf}}^{2}$*_r_*) and radius of gyration $R_{g,\mathrm{surf}}^{2}$, which can be seen plotted in Figure S3 in Supporting Information at *X_m_* = 0.7. In particular for this work, based on Equations (1)–(3) and upon the generation of a distribution of $R_{g,\mathrm{surf}}^{2}$, it is possible to assess the conformational character of the polymer layer in terms of the three regimes of conformation, i.e., mushroom, brush-like and brush [15]. The distribution of $R_{g,\mathrm{surf}}^{2}$ can be determined for the total population of polymer chains on the surface as well as for the two associated populations of dormant and dead polymer chains, as shown in Figure 8a–c considering *X_m_* = 0.7 and the reference conditions. It follows that for the reference $\theta_{R_{0,\mathrm{sur}}X}$ of 2.5 × 10^−1^ the total population of polymer chains and dormant chains on the surface (Figure 8a,b) are mainly represented by having a brush character (>80%), followed by a moderate percentage of polymer chains described as brush-like (≈18%), while only a very small portion of these polymer chains display a mushroom character (<1%). In contrast, Figure 8c shows that the largest portion of dead chains on the surface is best represented as having a brush-like character (≈60%), followed by about 27% of total dead chains, which adopt a mushroom conformation, while roughly 14% of these chains are considered to behave like brushes.

The evaluation of the conformational character of the individual polymer chains on the surface can be also performed as a function of *X_m_*, from which it is possible to follow the dynamic evolution of the excluded volume interactions between the chains in the polymer layer. Strong excluded volume interactions between the polymer chains promote an increase in the brush character, forcing the polymer chains to grow further away from the anchoring points on the surface, while weaker interactions are characterized by collapsed chains that extend in planes parallel to the surface, reflecting a larger mushroom character. Figure 8d–f show such evolutions for the total population of polymer chains, the dormant polymer chains and the dead chains on the surface. The low percentage of polymer chains behaving as mushroom, even at low *X_m_*, as can be seen in Figure 8d,e, indicates that the excluded volume interactions between the polymer chains are considerably important, which in turn is evident in a greater percentage of chains represented as brushes, especially at *X_m_* > 0.3. Conversely, Figure 8f shows that brush-like character is preferred for most dead chains on the surface, without significant changes of the proportionality between the three regimes as polymerization progresses. This is consistent with the results presented above, which allowed it to be established that the highest percentage of dead chains on the surface are formed during the first stages of polymerization, and these chains are characterized by having short chain lengths and, consequently, weaker excluded volume interactions in the polymer layer.

### 3.3. Effect of Average Initiator Surface Coverage on SI-RDRP Molecular Properties

In the previous subsections, the study of the SI-RDRP process was based on the reference $\theta_{R_{0,\mathrm{sur}}X}$ value of $2{.5\;\times \;10}^{-1}$. Here, the study is extended by introducing two additional values ($1{.0\;\times \;10}^{-1}$ and $5{.0\;\times \;10}^{-1}$) to evaluate the effect of $\theta_{R_{0,\mathrm{sur}}X}$ on the SI-RDRP molecular properties. The initial number of RDRP initiator molecules in both phases is still kept equal to 1 × 10^5^ to guarantee numerical accuracy, which imposes the need to modify the dimensions of the MC surface to model the respective new surface coverages.

**Figure 9:**
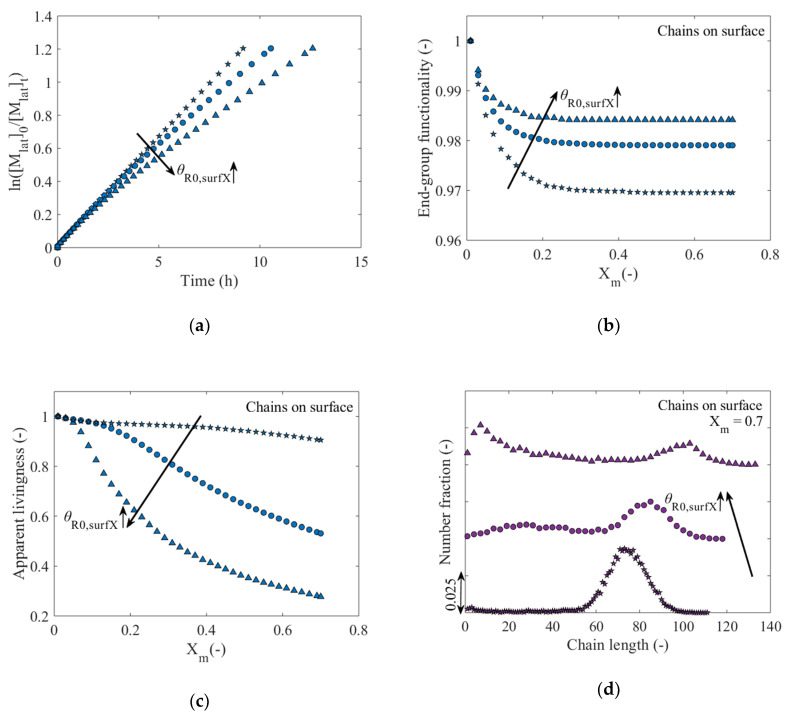

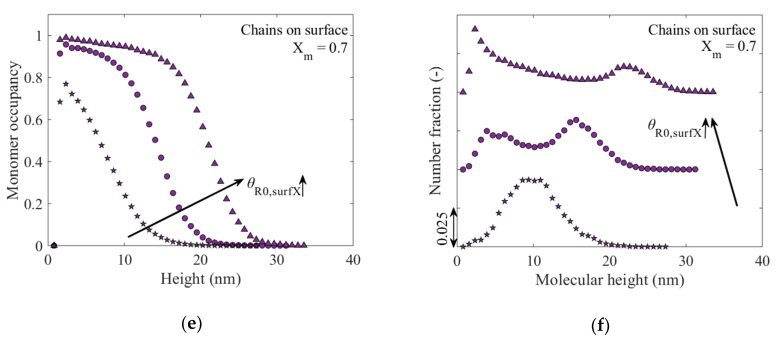
Effect of the average RDRP initiator surface coverage ($\theta_{R_{0,\mathrm{surg}}X}$) on (**a**) the evolution of ${\ln([M}_{\mathrm{lat}}{]}_{0}/{{[M}_{\mathrm{lat}}]}_{t})$ vs time, (**b**) the end-group functionality of surface-tethered dormant chains (*EGF_surf_*) as a function of monomer conversion (*X_m_*), (**c**) the apparent livingness (definition in Figure 5) of the polymer layer as a function of *X_m_*, (**d**) the chain length distribution (CLD) at *X_m_* = 0.7, (**e**) the monomer occupancy at *X_m_* = 0.7 and (**f**) the molecular height distribution (MHD) at *X_m_* = 0.7. Three different surface coverages are considered: $1{.0\;\times \;10}^{-1}$ (five-pointed stars), $2{.5\;\times \;10}^{-1}$ (circles), $5{.0\;\times \;10}^{-1}$ (upward-pointing triangles); simulation conditions: amount of RDRP initiator molecules $N_{R_{0,\mathrm{lat}}X}{=N}_{R_{0,\mathrm{surf}}X}$ = 1 × 10^5^, target chain length (TCL) of 100 in both phases (initial number of monomer molecules $N_{R_{0,\mathrm{lat}}X}$ = 2 × 10^7^); translational mode.

Figure 9a evaluates the effect of $\theta_{R_{0,\mathrm{sur}}X}$ on the evolution of ${\ln([M}_{\mathrm{lat}}{]}_{0}/{{[M}_{\mathrm{lat}}]}_{t})$ as a function of time up to *X_m_* = 0.7. It is observed that the polymerization process slows down as $\theta_{R_{0,\mathrm{sur}}X}$ increases. A closer inspection reveals that initially the three processes advance similarly but a deviation occurs around the second hour, from which the profiles of ${\ln([M}_{\mathrm{lat}}{]}_{0}/{{[M}_{\mathrm{lat}}]}_{t})$ for $\theta_{R_{0,\mathrm{sur}}X}=2{.5\;\times \;10}^{-1}$ (circles) and $\theta_{R_{0,\mathrm{sur}}X}=5{.0\;\times \;10}^{-1}$ (upward-pointing triangles) acquire a slightly downward curvature, while the corresponding profile for $\theta_{R_{0,\mathrm{sur}}X}=1{.0\;\times \;10}^{-1}$ (five-pointed stars) resembles a straight line as for a well-conducted SIP. Furthermore, the influence of $\theta_{R_{0,\mathrm{sur}}X}$ on the simulated *EGF_surf_* is shown in Figure 9b, in which it can be observed that a greater functionality is attained by increasing the surface coverage, which indicates that termination reactions leading to a decreased living/dormant character of the polymer layer are less favored at high values of $\theta_{R_{0,\mathrm{sur}}X}$. However, as revealed in Figure 9c, the increase in $\theta_{R_{0,\mathrm{sur}}X}$ results in a reduction in the apparent livingness of the polymer layer, since a greater proportion of dormant chains on the surface are hindered from further activation-growth-deactivation cycles due to stronger confinement on the surface (see Figure S4 in Supporting Information). The relevance of the introduced novel molecular property is thus further highlighted, as a conventional analysis based on only EGF_surf_ suggests the opposite trend. One thus needs to allow for some termination reactions to enable a controlled layer growth.

The influence of $\theta_{R_{0,\mathrm{sur}}X}$ on the chain length characteristics of the polymer layer is summarized in Figure 9d with focus on the CLDs of the total population of polymer chains on the surface at *X_m_* = 0.7. At a low $\theta_{R_{0,\mathrm{sur}}X}\;\mathrm{of}\;1{.0\;\times \;10}^{-1}$, a unimodal CLD results with a maximum just above a chain length of 70 (*x_n_* = 71; dispersity = 1.07; see in detail in Figure S5 in Supporting Information). In contrast, for $\theta_{R_{0,\mathrm{sur}}X}=2{.5\;\times \;10}^{-1}$ (*x_n_* = 63; dispersity = 1.19) and $\theta_{R_{0,\mathrm{sur}}X}=5{.0\;\times \;10}^{-1}$ (*x_n_* = 49; dispersity = 1.63), bimodal CLDs result. The first peak of the distribution is located in the low chain length region and increases with increasing $\theta_{R_{0,\mathrm{sur}}X}$. The second peak is located in the high chain length region and it is observed how the magnitude thereof decreases with increasing $\theta_{R_{0,\mathrm{sur}}X}$. These results clearly expose the strong influence of $\theta_{R_{0,\mathrm{sur}}X}$ with a decrease of *x_n_* and an increase of the dispersity with increasing $\theta_{R_{0,\mathrm{sur}}X}$ [15,44,50,53]. This in turn indicates that a greater confinement of the polymer chains on the surface due to an increase of $\theta_{R_{0,\mathrm{sur}}X}$ leads to a loss of control over the polymerization process, which can negatively and significantly alter the molecular properties of the polymer layer for specific applications.

Figure 9e explores the influence of $\theta_{R_{0,\mathrm{sur}}X}$ on the monomer occupancy profiles as a function of the lattice height in nm at *X_m_* = 0.7. It follows that the monomer occupancy profile broadens as $\theta_{R_{0,\mathrm{sur}}X}$ increases, which indicates an increase in the height of the polymer layer, since the higher level of confinement experienced by the chains on the surface forces them to grow in a more elongated way. In other words, the repulsions that are generated between the chains due to the lack of space on the surface result in a denser structure. Likewise, Figure 9f reveals the transition from a unimodal MHD for the total polymer chains on the surface for a low $\theta_{R_{0,\mathrm{sur}}X}$ to a bimodal MHD at higher $\theta_{R_{0,\mathrm{sur}}X}$, similar to the trend observed in the number CLDs (Figure 9d). This bimodality is mainly promoted by the increase in the percentage of hindered dormant polymer chains (Figure 9c) that cannot continue to propagate, thus being trapped within the polymer layer, consistent with the discussion of Figure 4f.

**Figure 10:**
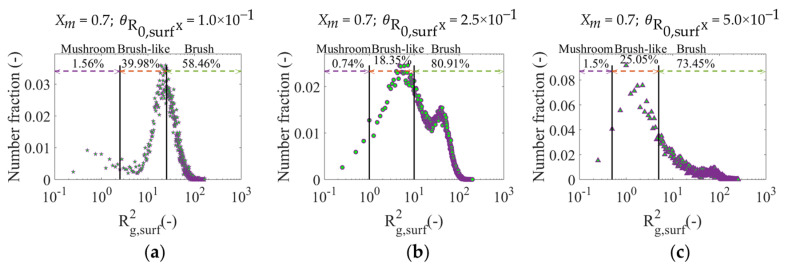
Total distribution of the squared radius of gyration ($R_{g,\mathrm{surf}}^{2}$) of individual surface-tethered polymer chains (purple-contoured full green symbols) grown on the surface at a monomer conversion $X_{m}$ = 0.7. Three (average) initiator surface coverages are considered: (**a**) $1{.0\;\times \;10}^{-1}$ (five-pointed star), (**b**) $2{.5\;\times \;10}^{-1}$ (circle), (**c**) $5{.0\;\times \;10}^{-1}$ (upward-pointing triangle). The two black vertical lines are calculated from Equations (1) and (3), and these lines are only there for a guide-of-the-eye; arrows denote bimodal character; conditions as in Figure 8; translational mode. Figure S6 in Supporting Information: evolution of the conformational regimes of the surface-tethered polymer chains as a function of monomer conversion.

With respect to the conformation qualification of the polymer layer on a chain by chain basis, Figure 10 shows the distribution of $R_{g,\mathrm{surf}}^{2}$ for the three $\theta_{R_{0,\mathrm{sur}}X}$ values under consideration. The bimodality observed in Figure 9d,f for the surface coverage of $2{.5\;\times \;10}^{-1}$ and $5{.0\;\times \;10}^{-1}\;$is also manifested in the $R_{g,\mathrm{surf}}^{2}$ distributions for these $\theta_{R_{0,\mathrm{sur}}X}$ values (Figure 10b,c). This is expected behavior as $R_{g,\mathrm{surf}}^{2}$ depends on the polymer chain length. The brush character is favored with the corresponding decrease in the percentage of chains on the surface that possess mushroom or brush-like conformation upon going from $\theta_{R_{0,\mathrm{sur}}X}$ of 1.0$\;{\times \;10}^{-2}$ to $2{.5\;\times \;10}^{-1}$. However, if $\theta_{R_{0,\mathrm{sur}}X}$ is again increased up to $5{.0\;\times \;10}^{-1}$, the percentage of chains in the brush regime decreases, since, as previously discussed, the formation of shorter (mushroom or brush-like) polymer chains is boosted due to an increased confinement at the surface.

### 3.4. Comparison of the Effect of the Translational and Segmental Diffusion Modes on SI-RDRP Properties

The above simulation results follow from modeling termination reactions between free and surface-tethered polymer chains based on the translational diffusion mode. This subsection explores the influence of the two modes of diffusion, i.e., the translational mode and the segmental mode, on the properties of the polymer layer obtained by performing an SI-RDRP process. For comparison purposes, we have also included simulation results obtained for a perfect SI-RDRP in which termination reactions are excluded.

**Figure 11:**
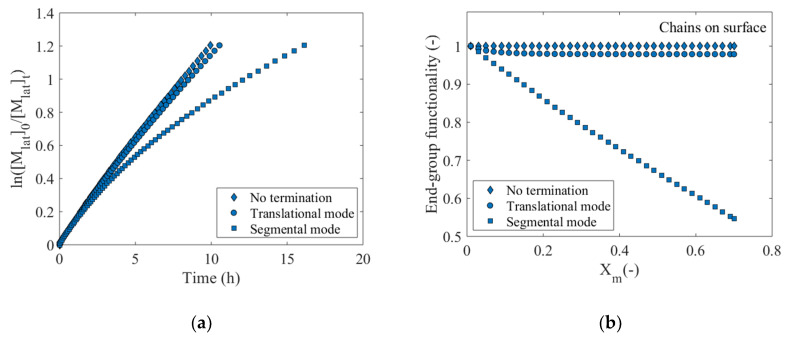

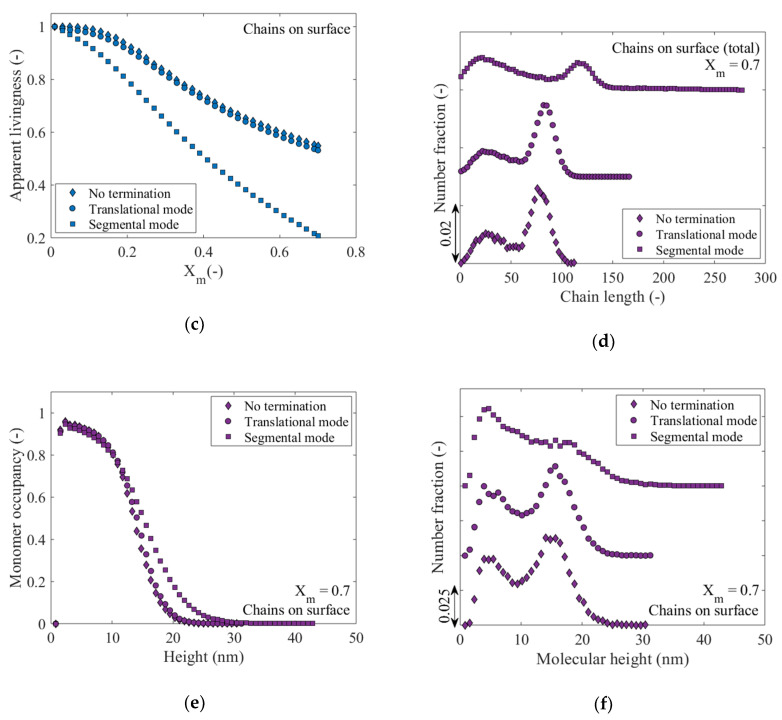
Theoretical evaluation of the effect of termination modes for solution-surface termination: (i) translational mode highlighting the case that a chain has to diffuse directly in an available space in the polymer layer (circles), (ii) segmental mode highlighting the case that a chain can alter locally its configuration in the available space (squares), as well as (iii) the reference case of absence of such termination reactions (diamonds) for the SI-RDRP process under conditions other than those in Figure 4 and parameters in Table 1. (**a**) ${\ln([M}_{\mathrm{lat}}{]}_{0}/{{[M}_{\mathrm{lat}}]}_{t})$ vs time. (**b**) End-group functionality of the surface-tethered polymer chains (*EGF_surf_*) and (**c**) apparent livingness of the polymer layer. (**d**) Number of chain length distributions of the surface-tethered polymer chains at *X_m_* = 0.7. (**e**) The monomer occupancies along the lattice height in nm at *X_m_* = 0.7 and (**f**) the molecular height distributions (MHDs) at *X_m_* = 0.7.

Figure 11a shows a comparison of ${\ln([M}_{\mathrm{lat}}{]}_{0}/{{[M}_{\mathrm{lat}}]}_{t})$ profiles as a function of time, obtained upon considering the translational diffusion mode (circles) and the segmental diffusion mode (squares), as well as the case of a polymerization process without termination (diamonds). All the simulations are performed with the reference $\theta_{R_{0,\mathrm{sur}}X}$ of 2.5 × 10^−1^, initial polymerization conditions as described in Section 2.2.2 and rate coefficients as shown in Table 1. As can be seen, the kinetic profiles corresponding to the polymerization case without termination reactions as well as the translational diffusion mode are very similar, with both processes achieving a monomer conversion of *X_m_* = 0.7 (${\ln([M}_{\mathrm{lat}}{]}_{0}/{{[M}_{\mathrm{lat}}]}_{t})\approx 1.2$) after approximately 10 h. In contrast, the SI-RDRP process slows down considerably after the implementation of the segmental diffusion mode in which *X_m_* = 0.7 is achieved after ca. 16 h, representing an approximate 60% increase in polymerization time. This slowdown is characterized by a marked downward curvature, originating from a strong increase of the number of successful termination reaction events. The latter is confirmed in Figure 11b in which it is observed that for the segmental diffusion mode *EGF_surf_* decreases to 0.55 at *X_m_* = 0.7, which contrasts sharply with the value of *EGF_surf_* = 0.98 at *X_m_* = 0.7 for the translational mode of diffusion (and the value of 1 for the case without termination reactions). In this context, a considerable difference between the values of the apparent livingness of the polymer layer is expected between both diffusion modes, as confirmed in Figure 11c. The apparent livingness of the polymer layer resulting from the segmental diffusion mode decreases more dramatically as a function of *X_m_* compared to the case of a translation diffusion mode, reaching values around 0.2 for the former and still over 0.5 for the latter, at *X_m_* = 0.7. The contribution of the hindered dormant chains is significant for both modes, with, e.g., a value just above 0.60 for the segmental mode and a value of 0.47 for the translational mode, which also results for the case in the absence of termination (Figure S7 in Supporting Information). Hence, the segmental mode is more severe for the increase of the relative formation of dead (scaling with all chains) and dormant hindered chains (scaling with respect to dormant chains only).

Figure 11d shows the influence of the termination diffusion mode on the number of CLDs generated from the total population of polymer chains on the surface at *X_m_* = 0.7. For completeness, Figure S8 in Supporting Information shows the variation of *x_n_* and dispersity of the different populations of polymer chains in both phases as a function of *X_m_*. The CLD corresponding to a polymerization in which termination reactions do not take place has been included for comparative purposes. In any case, a bimodal character results, although to a different degree. As can be seen, the CLD with no termination considered (*x_n_* = 61; dispersity = 1.17) as well as the CLD obtained from the translational diffusion mode (*x_n_* = 63; dispersity = 1.19) have considerable similarity to each other, although a long tail is visible for the latter, resulting from the formation of long dead polymer chains on the surface, slightly increasing *x_n_* and dispersity. As for the segmental diffusion mode, a more heterogeneous CLD results (*x_n_* = 69; dispersity = 1.49), which is characterized by an increase in the average chain length of the tethered chains (CLD positioned to the right) and a considerably greater dispersity compared to the translational mode. This distribution indicates loss of control over the SI-RDRP when the segmental mode is implemented, mainly due to the increase in the number of successful termination reactions between both phases. Unhindered dormant chains are forced to stretch and to grow significantly, but at the same time an unavoidable large bunch of hindered (shorter) dormant chains is still formed. Figure S9 in Supporting Information shows the evolution of the distributions of the total population of surface-tethered polymer chains as a function of *X_m_*, confirming this statement. Moreover, Figure S10 in Supporting Information shows the CLDs of the different chain populations either on the surface or in the solution at *X_m_* = 0.7 upon implementing the segmental mode. Following the implementation of the segmental mode, the total distribution is now influenced by both the dormant and dead CLD.

**Figure 12:**
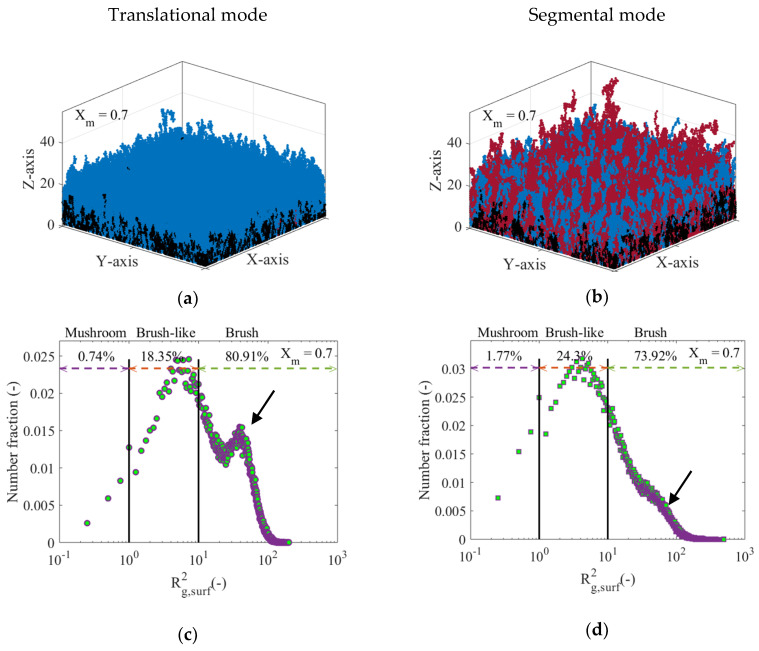
Snapshot of the polymer layer grown from RDRP initiator (*R*_0*,surf*_*X*) molecules chemically anchored on the flat surface with $\theta_{R_{0,\mathrm{sur}}X}$ = 0.25 for the two termination modes under consideration: (**a**) translational mode and (**b**) segmental mode; analogous plots for the total distribution of the squared radius of gyration ($R_{g,\mathrm{surf}}^{2}$) of individual surface-tethered polymer chains: (**c**) translational mode and (**d**) segmental mode. Same simulation conditions as in Figure 11. All the subplots are obtained at a monomer conversion *X_m_* = 0.7. Subplots (**a**,**c**) are the same as in Figure 7c and Figure 8.

The increase of the average chain length of the polymer chains on the surface arising from the segmental diffusion mode also promotes an increase in the height of the polymer layer, as shown in Figure 11e. Here, it is observed that the three monomer occupancy profiles as a function of the network height in nm at *X_m_* = 0.7 are almost identical up to a height of just over 10 nm, from which the profile related to the segmental diffusion mode becomes wider compared to the profiles for the other two cases. As revealed in Figure 11f, the MHD of the segmental diffusion mode is also wider and more heterogeneous than the other two MHDs, with a strong tail and pronounced fronting. The latter is visually represented in Figure 12a,b showing two snapshots of the polymer layer at *X_m_* = 0.7 obtained using the translational and segmental diffusion mode, respectively. Figure 12a shows a relatively homogeneous polymer layer highlighting the apparent lack of dead polymer chains (red lines), wherein only a few chains grow above the average layer height (same subfigure as Figure 7c). On the other hand, Figure 12b depicts a highly heterogeneous layer where a considerable portion of dead polymer chains (red chains) are observed, with some of them extending considerably beyond the average layer height, as reflected in the corresponding MHD in Figure 11f. Such chains are thus formed by termination between surface and solution species later on in the SI-RDRP at which point the polymer layer already has a certain thickness. More snapshots of the polymer layer at different *X_m_* have been included in Figure S11 in Supporting Information.

**Figure 13:**
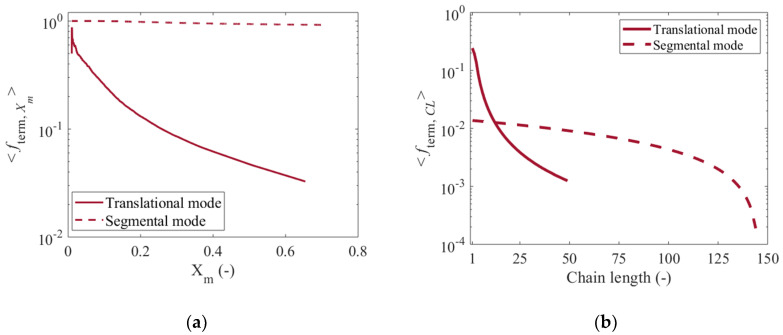
(**a**) Variation of the fraction of successful termination reaction events between surface and solution averaged for all the polymer chains <$f_{{\mathrm{term},X}_{m}}$ > as a function of monomer conversion (*X_m_*). (**b**) Variation of the fraction of successful termination reaction events averaged for all X*_m_* (<$f_{\mathrm{term},\mathrm{CL}}$>) as a function of (free solution) chain length. In both cases, a comparison of the two termination modes under consideration has been made. In addition, Figure S13 in the Supporting Information presents data related to termination by recombination and disproportion. Same conditions as in Figure 11 and Figure 12.

Figure 12c,d show the distributions of $R_{g,\mathrm{surf}}^{2}$ obtained after the explicit representation of the polymer layer from Figure 12a,b for the translational and the segmental diffusion mode, respectively. In the former subfigure (translational mode), a strongly dominant bimodal character is observed, while in the latter subfigure (segmental mode) this bimodality is still present, although less evident. This is a consequence of a reduction in the percentage of polymer chains that behave like brushes with a significant but not dominant role of hindered dormant chains. Figure S12 in Supporting Information shows the percentage variation of the regimes of conformation of the different populations of surface-tethered polymer chains as a function of *X_m_* for the two termination modes under consideration. With the segmental mode there is a more continuous formation of longer dormant chains, hindered (“shorter”) dormant chains and dead chains. The reduction in brush character can be further understood based on Figure 13a, which depicts the fraction of successful solution-surface termination reaction events averaged over all polymer chains as a function of *X_m_* (<$f_{{\mathrm{term},X}_{m}}$>). As can be seen, <$f_{{\mathrm{term},X}_{m}}$> drops drastically during the polymerization process upon implementing the translational mode (solid red line), whereas it only slightly decreases to approximately 0.92 at *X_m_* = 0.7 if the segmental mode is executed (dashed red line). For the segmental mode an increase in the fraction of shorter chains within the polymer layer is promoted, leading to the reduction of the fraction of polymer chains that can be represented as brushes, with a tendency toward a mushroom and brush-like character, as shown in Figure 12d.

The degree of success of the surface-solution termination reactions can also be evaluated as a function of the length of the polymer chains involved throughout the SI-RDRP. Figure 13b therefore shows the fraction of successful termination reaction events averaged over all *X_m_* values (<$f_{\mathrm{term},\mathrm{CL}}$>) as a function of the chain length of the free polymer chains in the solution near the surface. In particular, a sharp decrease in <$f_{\mathrm{term},\mathrm{CL}}$> is observed as the chain length increases if the translational mode is implemented (solid red line). Consistent with translational diffusion theory for polymers [73,74], it follows that the diffusion of a free polymer chain in the solution to an active/dormant center in the polymer layer becomes much more difficult as the chain length increases. (Direct) translational diffusion can only be carried out up to a certain maximum chain length, approximately 50 under the reference conditions, after which all translational mode termination attempts fail. As for the segmental mode (dashed red line), the observed behavior can be divided into two main regions. From the mildly decreasing trend shown in the first region, which goes from a chain length of 1 to about 100, it can be deduced that the termination reactions between both phases occur with a relatively high probability of success regardless of the length of the free polymer chain that diffuses into the polymer layer. There is thus enough space in the polymer layer so that the chain can be segment-wise relocated. However, for polymer chains in solution with chain lengths larger than 100 (second region) a rapid decrease in the fraction of successful termination events is observed, indicating that the diffusion of the free chains into the polymer layer as well as their local rearrangement become more arduous, hence, the associated termination rate declines. Such larger chain lengths are formed at larger *X_m_* in a RDRP mechanism. Combined with less space, it can be understood why too long chains fail in realizing the termination event even if their monomer units are allowed to move segment-wise.

Figure 10, Figure 11, Figure 12, Figure 13 thus highlight that a better understanding of the dominant diffusion mode should be targeted in future SI-RDRP kinetic studies. Notably different responses on the level of the distribution are associated with both modes, potentially opening the pathway regarding the determination of their relative importance upon combining the current *k*MC platform with experimental analysis tools.

## 4. Conclusions

The developed matrix-based *k*MC tool based on an implicit mechanism to study a general SI-RDRP provides valuable information on the molecular characteristics of polymer chains grown from flat surfaces, which are difficult to access only through experimental analysis. Moreover, the molecular characteristics of the surface-tethered polymer chains can be compared with those of the polymer chains that are free in the near-surface solution, highlighting differences as a consequence of the dissimilarities of the reaction environments.

It is demonstrated that the confinement kinetic effect for the surface-tethered polymer chains promotes the formation of a polymeric layer made up of shorter and more heterogeneously composed polymer chains compared to the free chains in solution. Specifically, bimodality results in the number of CLDs and the MHD, a new distribution introduced in the present work. This bimodality can be explained by a significant to dominant role for shorter hindered dormant polymer chains. Such chains are incapable of further activation-growth-deactivation cycles and need to be considered to quantify the success of a SI-RDRP. An apparent livingness is therefore put forward in the present work, penalizing not only dead chains formed via termination but also hindered dormant chains that have no direct space around the dormant moiety to enable further modification.

The matrix-based *k*MC model also allows access of the detailed 3D composition of each of the chains on the surface as well as their individual classification based on the regimes of conformation. This enables a more suitable description of the polymer layer in relation to the percentage of chains that have a mushroom, brush-like or brush character, still differentiating between their dormant and dead nature. It follows that dead chains have a less brush character, but with controlled SI-RDRP conditions one can mainly focus on the dormant chains for which the brush character is higher. Here, an important parameter is the initial average surface coverage, with a higher one giving rise to less dead chains but also a lower apparent livingness. The monomer occupancy and maximal layer height increase as well but also more hindered shorter dormant species are formed. A complex relation of reaction conditions and SI-RDRP performance thus results, and it is clear that an advanced *k*MC model facilitates the selection of experimental conditions with the aim of adequately designing SI-RDRP processes that can lead to the desired macroscopic properties for specific applications.

Future work should be directed regarding the reliable determination of SI-RDRP-specific kinetic parameters and an extension regarding interactions between surface and anchored species on the one hand and mutual interactions between anchored species on the other hand beyond excluded volume interactions. A specific task is also the concrete quantification of the solution-surface termination behavior. The current work has demonstrated that two extreme termination modes (translational and segmental) have a significant effect on the molecular properties of the resulting polymer layer. The translational mode resembles a process closer to a living polymerization system in which termination reactions are not allowed, while the segmental mode favors a considerable increase in the number of termination reactions, which is why it resembles a process in which the molecular control is more reduced. Furthermore, the current work highlights that both diffusion modes are chain length dependent opening the pathway to the development of fundamental diffusion laws for surface-based systems.

## Figures and Tables

**Table 1:** Lumped chemical reactions to model surface-initiated reversible deactivation radical polymerization (SI-RDRP; formally case of normal SI-ATRP) by means of kinetic Monte Carlo (kMC) simulations. Also given are the lumped apparent rate coefficients ($k_{j,k}^{\mathrm{app}}$) and the associated elementary rate coefficients ($k_{j,k}$) to calculate these $k_{j,k}^{\mathrm{app}}$ values; *j*: reaction type; *k*; phase (surf, lat, lat-surf); the equilibrium coefficient ($K_{\mathrm{eq}})$ for the calculation of $k_{j,k}^{\mathrm{app}}$ values is for simplicity always 1.5$\times 10^{-6}$; for reactions on the surface the actual MC rate in s^−1^ (Equations (14)–(16)) is further corrected as described in Section 2.2 to take into account the impact of confinement on the kinetics (availability of space and reactive center(s) at reaction distance). $k_{j,k}$ values used in this work are based on the polymerization of methyl methacrylate at 355 K.

Lumped Reaction	Reaction Equation	$k_{j,k}^{\mathrm{app}}\;({\mathrm{dm}}^{n}{\mathrm{mol}}^{-1}s^{-1})$	$k_{j,k}\;({\mathrm{dm}}^{n}{\mathrm{mol}}^{-1}s^{-1})$	*n ^a^*	Ref
Chain initiation	$R_{0,\mathrm{surf}}{X+M}_{\mathrm{lat}}\overset{k_{i,\mathrm{lat}\text{-}\mathrm{surf}}^{\mathrm{app}}}{\to }R_{1,\mathrm{surf}}X$	$k_{i,\mathrm{lat}\text{-}\mathrm{surf}}^{\mathrm{app}}{=K}_{\mathrm{eq}}{\times k}_{i,\mathrm{lat}\text{-}\mathrm{surf}}$	$k_{i,\mathrm{lat}\text{-}\mathrm{surf}}=1{.1\;\times \;10}^{6}$ ^c^	3	[58]
	$R_{0,\mathrm{lat}}{X+M}_{\mathrm{lat}}\overset{k_{i,\mathrm{lat}}^{\mathrm{app}}}{\to }R_{1,\mathrm{lat}}X$	$k_{i,\mathrm{lat}\;}^{\mathrm{app}}{=K}_{\mathrm{eq}}{\times k}_{i,\mathrm{lat}}$	$k_{i,\mathrm{lat}\;}=1{.1\;\times \;10}^{6}$	3	[58]
Propagation	$R_{i,\mathrm{surf}}{X+M}_{\mathrm{lat}}\overset{k_{p,\mathrm{lat}\text{-}\mathrm{surf}}^{\mathrm{app}}}{\to }R_{i+1,\mathrm{surf}}X$	$k_{p,\mathrm{lat}\text{-}\mathrm{surf}}^{\mathrm{app}}{=K}_{\mathrm{eq}}{\times k}_{p,\mathrm{lat}\text{-}\mathrm{surf}}$	$k_{p,\mathrm{lat}\text{-}\mathrm{surf}\;}=1{.3\;\times \;10}^{3}$ *^c^*	3	[58]
	$R_{i,\mathrm{lat}}{X+R}_{j,\mathrm{lat}}X\overset{k_{\mathrm{td},\mathrm{lat}}}{\to }P_{i,\mathrm{lat}}{+P}_{j,\mathrm{lat}}$	$k_{p,\mathrm{lat}}^{\mathrm{app}}{=K}_{\mathrm{eq}}{\times k}_{p,\mathrm{lat}}$	$k_{p,\mathrm{lat}\;}=1{.3\;\times \;10}^{3}$	3	[58]
Termination by recombination	$R_{i,\mathrm{surf}}{X+R}_{j,\mathrm{surf}}X\overset{k_{\mathrm{tc},\mathrm{surf}}^{\mathrm{app}}}{\to }P_{i+j,\mathrm{surf}}$	$k_{\mathrm{tc},\mathrm{surf}}^{\mathrm{app}}{=K}_{\mathrm{eq}}{}^{2}{\times k}_{\mathrm{tc},\mathrm{surf}}$	$k_{\mathrm{tc},\mathrm{surf}\;}=7{.3\;\times \;10}^{14}$	2	This work
	$R_{i,\mathrm{surf}}{X+R}_{j,\mathrm{lat}}X\overset{k_{\mathrm{tc},\mathrm{lat}\text{-}\mathrm{surf}}^{\mathrm{app}}}{\to }P_{i+j,\mathrm{surf}}$	$k_{\mathrm{tc},\mathrm{lat}\text{-}\mathrm{surf}}^{\mathrm{app}}{=K}_{\mathrm{eq}}{}^{2}{\times k}_{\mathrm{tc},\mathrm{lat}\text{-}\mathrm{surf}}$	$k_{\mathrm{tc},\mathrm{lat}\text{-}\mathrm{surf}}=5{.0\;\times \;10}^{8}$ *^c^*	3	[59]
	$R_{i,\mathrm{lat}}{X+R}_{j,\mathrm{lat}}X\overset{k_{\mathrm{tc},\mathrm{lat}}^{\mathrm{app}}}{\to }P_{i+j,\mathrm{lat}}$	$k_{\mathrm{tc},\mathrm{lat}}^{\mathrm{app}}{=K}_{\mathrm{eq}}{}^{2}{\times k}_{\mathrm{tc},\mathrm{lat}}$	$k_{\mathrm{tc},\mathrm{lat}}=5{.0\;\times \;10}^{8}$ *^b^*	3	[59]
Termination by disproportionation	$R_{i,\mathrm{surf}}{X+R}_{j,\mathrm{surf}}X\overset{k_{\mathrm{td},\mathrm{surf}}^{\mathrm{app}}}{\to }P_{i,\mathrm{surf}}{+P}_{j,\mathrm{surf}}$	$k_{\mathrm{td},\mathrm{surf}}^{\mathrm{app}}{=K}_{\mathrm{eq}}{}^{2}{\times k}_{\mathrm{td},\mathrm{surf}}$	$k_{\mathrm{td},\mathrm{surf}}=1{.1\;\times \;10}^{15}$	2	This work
	$R_{i,\mathrm{surf}}{X+R}_{j,\mathrm{lat}}X\overset{k_{\mathrm{td},\mathrm{lat}\text{-}\mathrm{surf}}^{\mathrm{app}}}{\to }P_{i,\mathrm{surf}}{+P}_{j,\mathrm{lat}}$	$k_{\mathrm{td},\mathrm{lat}\text{-}\mathrm{surf}}^{\mathrm{app}}{=K}_{\mathrm{eq}}{}^{2}{\times k}_{\mathrm{td},\mathrm{lat}\text{-}\mathrm{surf}}$	$k_{\mathrm{td},\mathrm{lat}\text{-}\mathrm{surf}}=7{.6\;\times \;10}^{8}$ *^c^*	3	[59]
	$R_{i,\mathrm{lat}}{X+R}_{j,\mathrm{lat}}X\overset{k_{\mathrm{td},\mathrm{lat}}}{\to }P_{i,\mathrm{lat}}{+P}_{j,\mathrm{lat}}$	$k_{\mathrm{td},\mathrm{lat}}^{\mathrm{app}}{=K}_{\mathrm{eq}}{}^{2}{\times k}_{\mathrm{td},\mathrm{lat}}$	$k_{\mathrm{td},\mathrm{lat}}=7{.6\;\times \;10}^{8}$ *^b^*	3	[59]

*^a^* The value of *n* depends on whether the reactions take place only on the surface (*n* = 2) or if the reactions occur in the solution or between the solution and the surface (*n* = 3). *^b^* The values correspond to the termination rate coefficients between two unimers, as reported by Johnston-Hall and Monteiro [59]. *^c^* For simplicity, the nominal values of the elementary rate coefficients for chain initiation and propagation reactions on the surface ($k_{i,\mathrm{lat}\text{-}\mathrm{surf}}\;$and $k_{p,\mathrm{lat}\text{-}\mathrm{surf}\;}$) as well as termination reactions between surface-tethered and solution free macrospecies ($k_{\mathrm{tc},\mathrm{lat}\text{-}\mathrm{surf}}\;$and $k_{\mathrm{td},\mathrm{lat}\text{-}\mathrm{surf}}$) adopt the same values as those in the lattice/solution near the surface, based on the work of Zhu and co-workers [47,48,52,60].

## Supplementary Materials

The supplementary materials are available online at https://www.mdpi.com/2073-4360/12/6/1409/s1. Figure S1: Study of the surface-initiated reversible deactivation radical polymerization process under reference conditions as a function of monomer conversion *X_m_* (Figure 4 in the main text): (a) reaction probabilities; (b) variation of the number of chemical species in the reaction system. Figure S2: Number average chain length distributions of the different populations of polymer chains in the SI-RDRP process as a function of monomer conversion. Top: (a) total population of polymer chains on surface; (b) dormant chains on surface; (c) dead chains on surface. Bottom: (d) total population of free polymer chains in lattice; (e) free dormant polymer chain in lattice; (f) free dead chains in lattice. Reference conditions as in Figure S1. Figure S3: (a) Log-log plot of the conformational characteristics of the surface-tethered polymer chains, either dormant or dead, represented by the mean-square end-to-end distance ${<R}_{e,\mathrm{surf}}^{2}>$ and radius of gyration ${<R}_{g,\mathrm{surf}}^{2}>$ as a function of chain length. (b) Representation of $R_{e,\mathrm{surf}}^{2}$ versus $R_{g,\mathrm{surf}}^{2}$ of the surface-tethered polymer chains at a monomer conversion *X_m_* = 0.7. (c) From the data in b, ${<R}_{e,\mathrm{surf}}^{2}>$ is represented as a function of ${<R}_{g,\mathrm{surf}}^{2}>$ (values per chain length). The solid honey yellow line depicts the expected scaling behavior for free chains: $R_{e,\mathrm{surf}}^{2}$⁄$R_{g,\mathrm{surf}}^{2}$ = 6. In these plots, the properties of the surface-tethered dormant chains are depicted in blue (blue-contoured circles in (a) and solid blue circles in (b) and (c)) while the properties of the surface-tethered dead chains are depicted in red (red-contoured circles in (a) and solid red circles in (b) and (c)). Same reference conditions are in Figures S1 and S2. Figure S4: Study of the influence of the average RDRP initiator surface coverage ($\theta_{R_{0,\mathrm{sur}}X}$) on the evolution of the percentage of surface-tethered dormant polymer chains that become hindered from activation-growth-deactivation within the polymer layer due to shielding as a function of monomer conversion (*X_m_*). Three different surface coverage are considered: $1{.0\;\times \;10}^{-1}$ (five-pointed stars), $2{.5\;\times \;10}^{-1}$ (circles), $5{.0\;\times \;10}^{-1}$ (upward-pointing triangles). Same reference conditions as in Figure 9 in the main text. Figure S5: Study of the influence of the average RDRP surface coverage ($\theta_{R_{0,\mathrm{sur}}X}$) on the molecular properties of the polymerization products as a function of monomer conversion (*X_m_*). Number average chain length (*x_n_*; top figures) and dispersity (bottom figures): (a) and (d) $1{.0\;\times \;10}^{-1}$ (five-pointed stars); (b) and (e) $2{.5\;\times \;10}^{-1}$ (circles); (c) and (f) $5{.0\;\times \;10}^{-1}$ (upward-pointing triangles). Free polymer chains are represented as contoured empty symbols while surface-tethered polymer chains are represented as solid symbols. Total population (purple), dormant chains (blue) and dead chains (red). Same reference conditions as in Figure S4. Figure S6: Variation of the percentage of the surface-tethered polymer chains as a function of monomer conversion (*X_m_*) with mushroom (honey yellow), brush-like (orange) or brush (green) conformation for the three average RDRP initiator surface coverages. $1{.0\times 10}^{-1}$ (five-pointed stars), $2{.5\;\times \;10}^{-1}$ (circles), $5{.0\;\times \;10}^{-1}$ (upward-pointing triangles). Color of the outline of the different symbols used: total population (purple), dormant chains (blue) and dead chains (red). Same reference conditions as in Figures S4 and S5. Figure S7: Study of the influence of the termination mode on the evolution of the percentage of surface-tethered dormant polymer chains that become hindered from propagation within the polymer layer due to shielding as a function of monomer conversion (*X_m_*). i) Translational mode highlighting the case that a chain has to diffuse directly in an available space in the polymer layer (circles); (ii) Segmental mode highlighting the case that a chain can alter locally its configuration in the available space (squares) as well as (iii) the reference case of absence of such termination reactions (diamonds) for the SI-RDRP process. Reference conditions as in Figure 11 in main text. Figure S8: Study of the influence of the termination mode on the molecular properties of the polymerization products as a function of monomer conversion (*X_m_*). Number average chain length (*x_n_*; top figures) and dispersity (bottom figures): (a) and (d) Absence of termination (diamond); (b) and (e) Translational mode (circles); (c) and (f) segmental mode (squares). Free polymer chains are represented as contoured empty symbols while surface-tethered polymer chains are represented as solid symbols. Total population (purple), dormant chains (blue) and dead chains (red). Same reference conditions as in Figure S7. Figure S9: Number average chain length distributions of the total population of surface-tethered polymer chains in the SI-RDRP process as a function of monomer conversion. (a) Reference case of absence of termination reactions; (b) translational mode; (c) segmental mode. Reference conditions as in Figure S7. Figure S10: Top: Number chain length distribution of (a) all surface-tethered polymer chains (black-contoured full purple circles); (b) surface-tethered dormant chains (black-contoured full blue circles) and (c) surface-tethered dead polymer chains (black-contoured full red circles). Bottom: Number chain length distribution of (d) all free polymer chains (purple-contoured empty circles) (e) free dormant chains (blue-contoured empty circles) and (f) free dead polymer chains (red-contoured empty circles). In (a) and (d) the regions circled in red represent the contributions of dead polymer chains to the CLD of the total polymer species in both phases. These distributions are obtained at a monomer conversion *X_m_* = 0.7. Same reference conditions (segmental mode) conditions as in Figure S7. Figure S11: Snapshot of the polymer layer grown for two termination modes. The snapshots are taken a three different monomer conversion: (a) and (b) *X_m_* = 0.1; (c) and (d) *X_m_* = 0.4; (e) and (f) *X_m_* = 0.7. Surface-tethered dormant chains are represented in blue, dead chains on the surface are represented in red while the hindered dormant chains that cannot continue to propagate by the RDPR mechanism due to shielding are represented in black. Left column: translational mode; right column: segmental mode. Same reference conditions as in Figures S8 and S9. Figure S12: Variation of the percentage of the surface-tethered polymer chains as a function of monomer conversion (*X_m_*) with mushroom (honey yellow), brush-like (orange) or brush (green) conformation for the two termination modes under consideration. Left column: translational mode (circles); Right column: segmental mode (squares). Color of the outline of the different symbols used: total population (purple), dormant chains (blue) and dead chains (red). Same reference conditions as in Figures S8–S10. Figure S13: Variation of the fraction of successful termination reaction events averaged for all the polymer chains <$f_{{\mathrm{term},X}_{m}}$> as a function of monomer conversion: (a) termination by recombination (red); (b) termination by disproportionation (honey yellow). Variation of the fraction of successful termination reaction events averaged for all monomer conversion <$f_{\mathrm{term},\mathrm{CL}}$> as a function of chain length: (c) termination by recombination (red); (d) termination by disproportionation (honey yellow). For all cases, a comparison of the two termination modes under consideration has been made. Same reference conditions as in Figures S8–S11.
