# Supplementary material for: The Competition of Termination and Shielding to Evaluate the Success of Surface-Initiated Reversible Deactivation Radical Polymerization
Source: Polymers (Basel). 2020 Jun 23;12(6):1409. doi: 10.3390/polym12061409 (PMC7361790; doi:10.3390/polym12061409)
Supplement: Supplementary file 1 [file polymers-12-01409-s001.pdf]

Supporting Information

# Supporting information "The competition of termination and shielding to evaluate the success of surface-initiated reversible deactivation radical polymerization"

**Francisco J. Arraez [1], Paul H. M. Van Steenberge [1] and Dagmar R. D'hooge [1,2,*]**

[1] Laboratory for Chemical Technology, Department of Materials, Textiles and Chemical Engineering, Ghent University, Technologiepark 125, Zwijnaarde, Ghent 9052, Belgium.
[2] Centre for Textile Science and Engineering, Department of Materials, Textiles and Chemical Engineering, Ghent University, Technologiepark 70A, Zwijnaarde, Ghent 9052, Belgium.
* Correspondence: dagmar.dhooge@ugent.be

Received: date; Accepted: date; Published: date

**Contents**

Extra information for Subsection 3.1 ........ 2
Extra information for Subsection 3.2 ........ 3
Extra information for Subsection 3.3 ........ 5
Extra information for Subsection 3.4 ........ 8
References ........ 13

## Extra information for Subsection 3.1

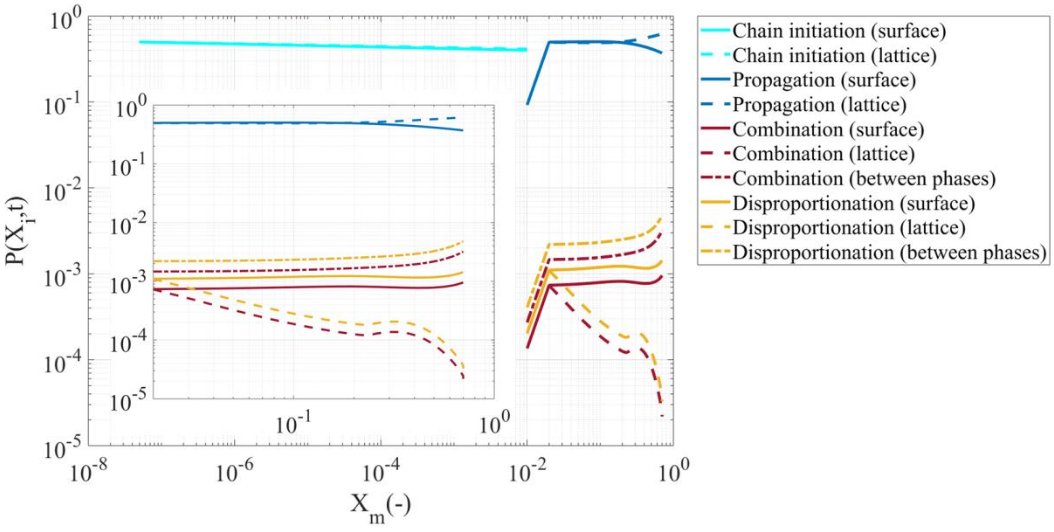


(**a**)

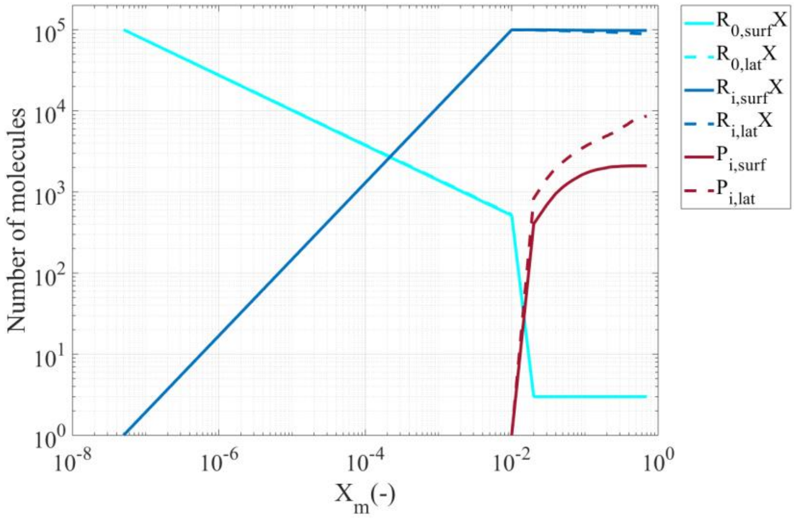


(**b**)

**Figure S1.** Study of the surface-initiated reversible deactivation radical polymerization process under reference conditions as a function of monomer conversion $X_m$ (**Figure 4** in the main text): (a) reaction probabilities; (b) variation of the number of chemical species in the reaction system.

## Extra information for Subsection 3.2

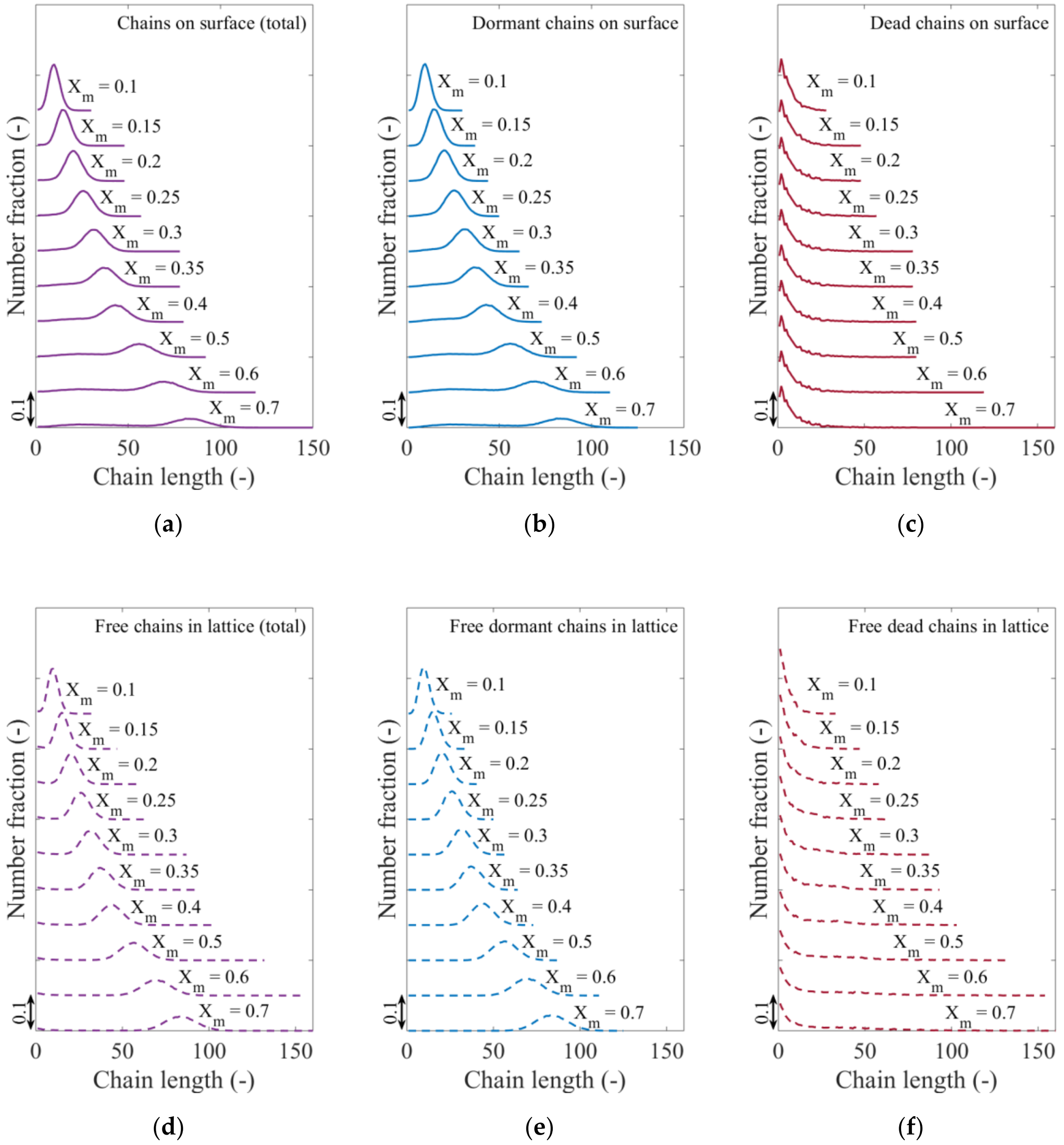


**Figure S2.** Number average chain length distributions of the different populations of polymer chains in the SI-RDRP process as a function of monomer conversion. Top: (a) total population of polymer chains on surface; (b) dormant chains on surface; (c) dead chains on surface. Bottom: (d) total population of free polymer chains in lattice; (e) free dormant polymer chain in lattice; (f) free dead chains in lattice. Reference conditions as in **Figure S1**.

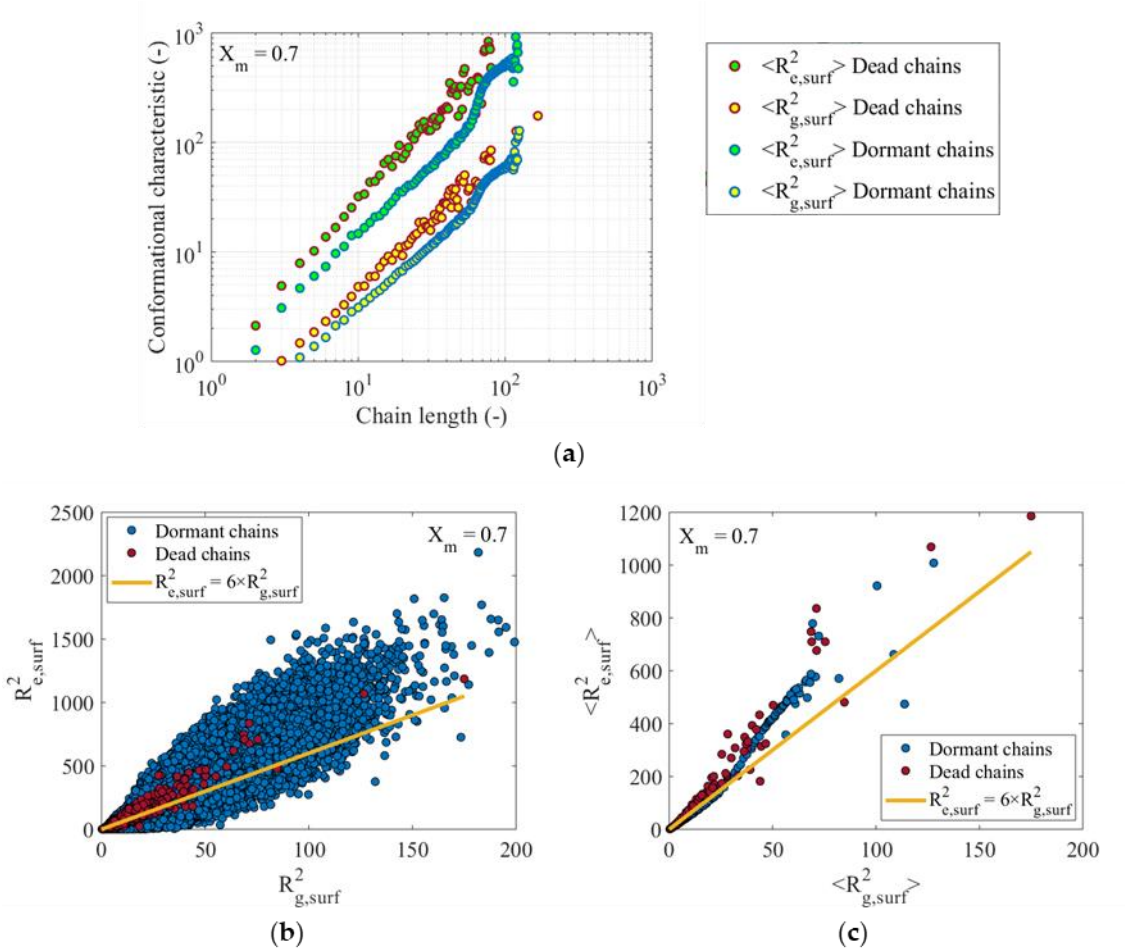


**Figure S3.** (a) Log-log plot of the conformational characteristics of the surface-tethered polymer chains, either dormant or dead, represented by the mean-square end-to-end distance $<R^2_{e,surf}>$ and radius of gyration $<R^2_{g,surf}>$ as a function of chain length. (b) Representation of $R^2_{e,surf}$ versus $R^2_{g,surf}$ of the surface-tethered polymer chains at a monomer conversion $X_m$ = 0.7. (c) From the data in b, $<R^2_{e,surf}>$ is represented as a function of $<R^2_{g,surf}>$ (values per chain length). The solid honey yellow line depicts the expected scaling behavior for free chains: $R^2_{e,surf}/R^2_{g,surf}$ = 6[1, 2]. In these plots, the properties of the surface-tethered dormant chains are depicted in blue (blue-contoured circles in (a) and solid blue circles in (b) and (c)) while the properties of the surface-tethered dead chains are depicted in red (red-contoured circles in (a) and solid red circles in (b) and (c)). Same reference conditions are in **Figure S1 and S2.**

**Extra information for Subsection 3.3**

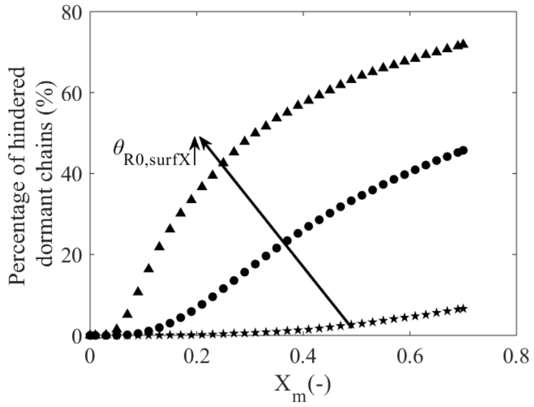


**Figure S4.** Study of the influence of the average RDRP initiator surface coverage ($\theta_{\mathrm{R0,sur}}\mathrm{X}$) on the evolution of the percentage of surface-tethered dormant polymer chains that become hindered from activation-growth-deactivation within the polymer layer due to shielding as a function of monomer conversion ($X_m$). Three different surface coverage are considered: $1.0\times10^{-1}$ (five-pointed stars), $2.5\times10^{-1}$ (circles), $5.0\times10^{-1}$ (upward-pointing triangles). Same reference conditions as in **Figure 9** in the main text.

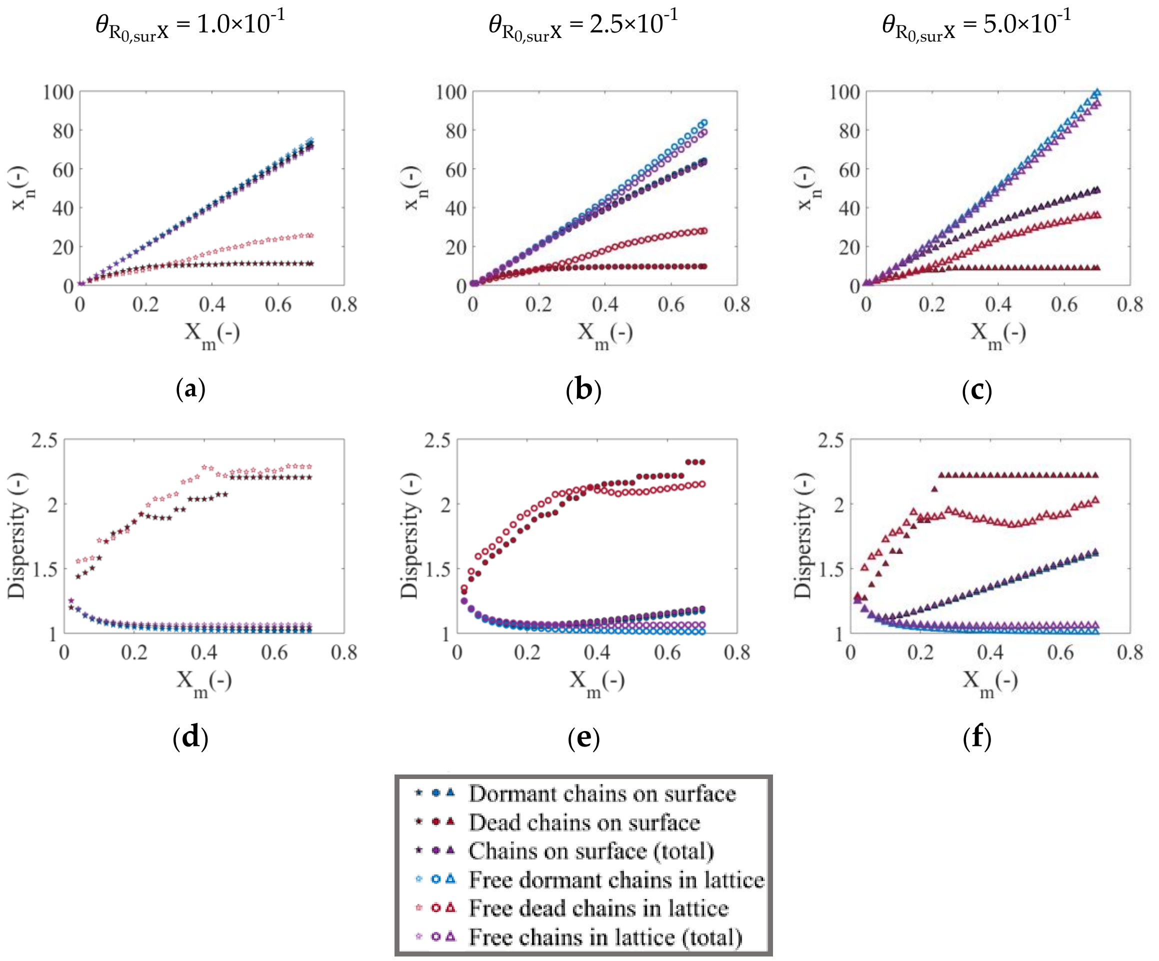


**Figure S5.** Study of the influence of the average RDRP surface coverage ($\theta_{R_{0,sur}X}$) on the molecular properties of the polymerization products as a function of monomer conversion ($X_m$). Number average chain length ($x_n$; top figures) and dispersity (bottom figures) : (a) and (d) $1.0\times10^{-1}$ (five-pointed stars); (b) and (e) $2.5\times10^{-1}$ (circles); (c) and (f) $5.0\times10^{-1}$ (upward-pointing triangles). Free polymer chains are represented as contoured empty symbols while surface-tethered polymer chains are represented as solid symbols. Total population (purple), dormant chains (blue) and dead chains (red). Same reference conditions as in **Figure S4**.

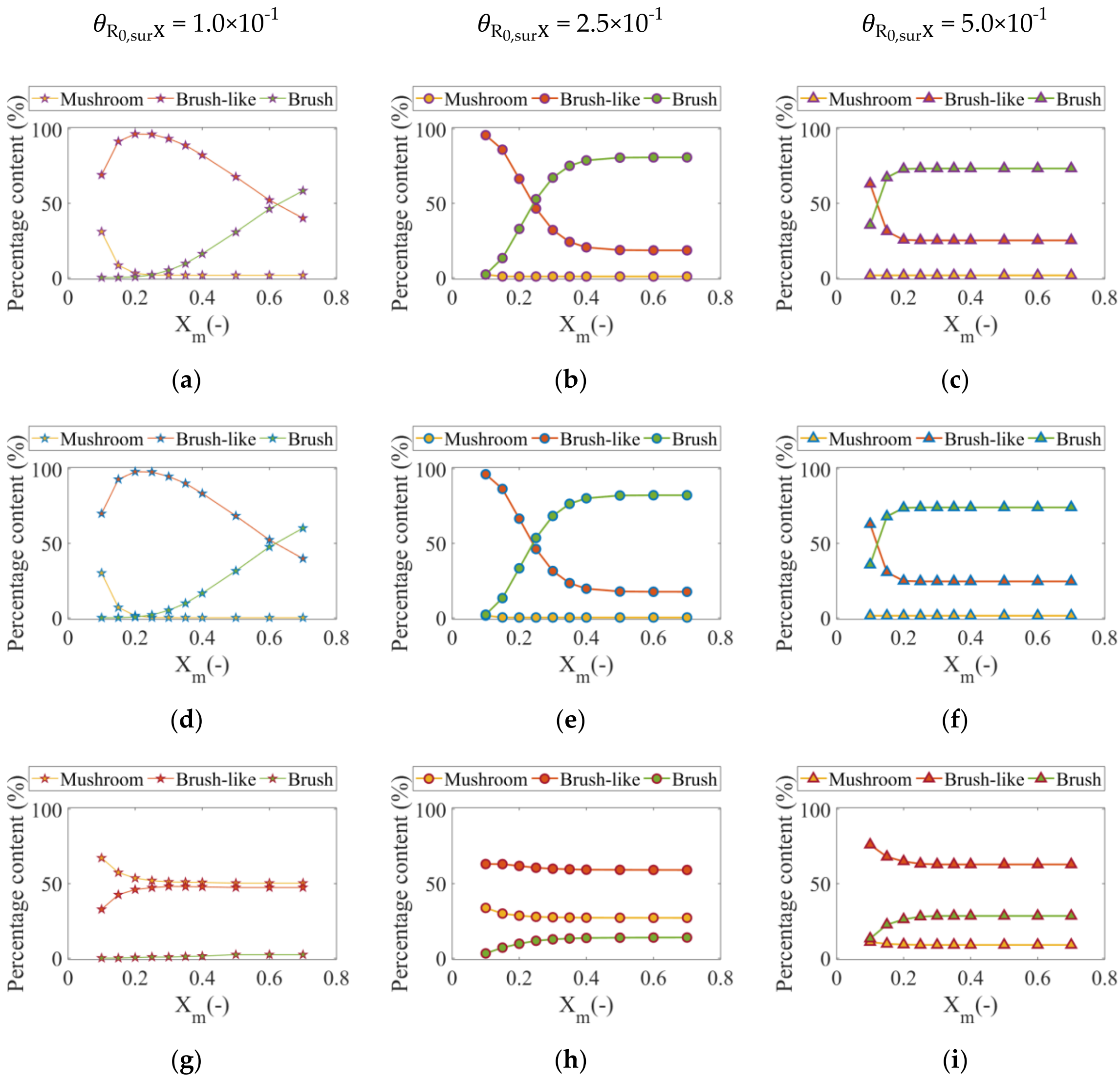


**Figure S6.** Variation of the percentage of the surface-tethered polymer chains as a function of monomer conversion ($X_m$) with mushroom (honey yellow), brush-like (orange) or brush (green) conformation for the three average RDRP initiator surface coverages. 1.0×10$^{-1}$ (five-pointed stars), 2.5×10$^{-1}$ (circles), 5.0×10$^{-1}$ (upward-pointing triangles). Color of the outline of the different symbols used: total population (purple), dormant chains (blue) and dead chains (red). Same reference conditions as in **Figure S4** and **S5**.

**Extra information for Subsection 3.4**

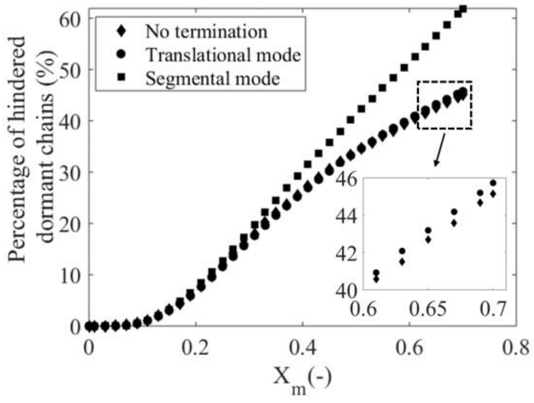


**Figure S7.** Study of the influence of the termination mode on the evolution of the percentage of surface-tethered dormant polymer chains that become hindered from propagation within the polymer layer due to shielding as a function of monomer conversion ($X_m$). i) Translational mode highlighting the case that a chain has to diffuse directly in an available space in the polymer layer (circles); (ii) Segmental mode highlighting the case that a chain can alter locally its configuration in the available space (squares) as well as (iii) the reference case of absence of such termination reactions (diamonds) for the SI- RDRP process. Reference conditions as in **Figure 11** in main text.

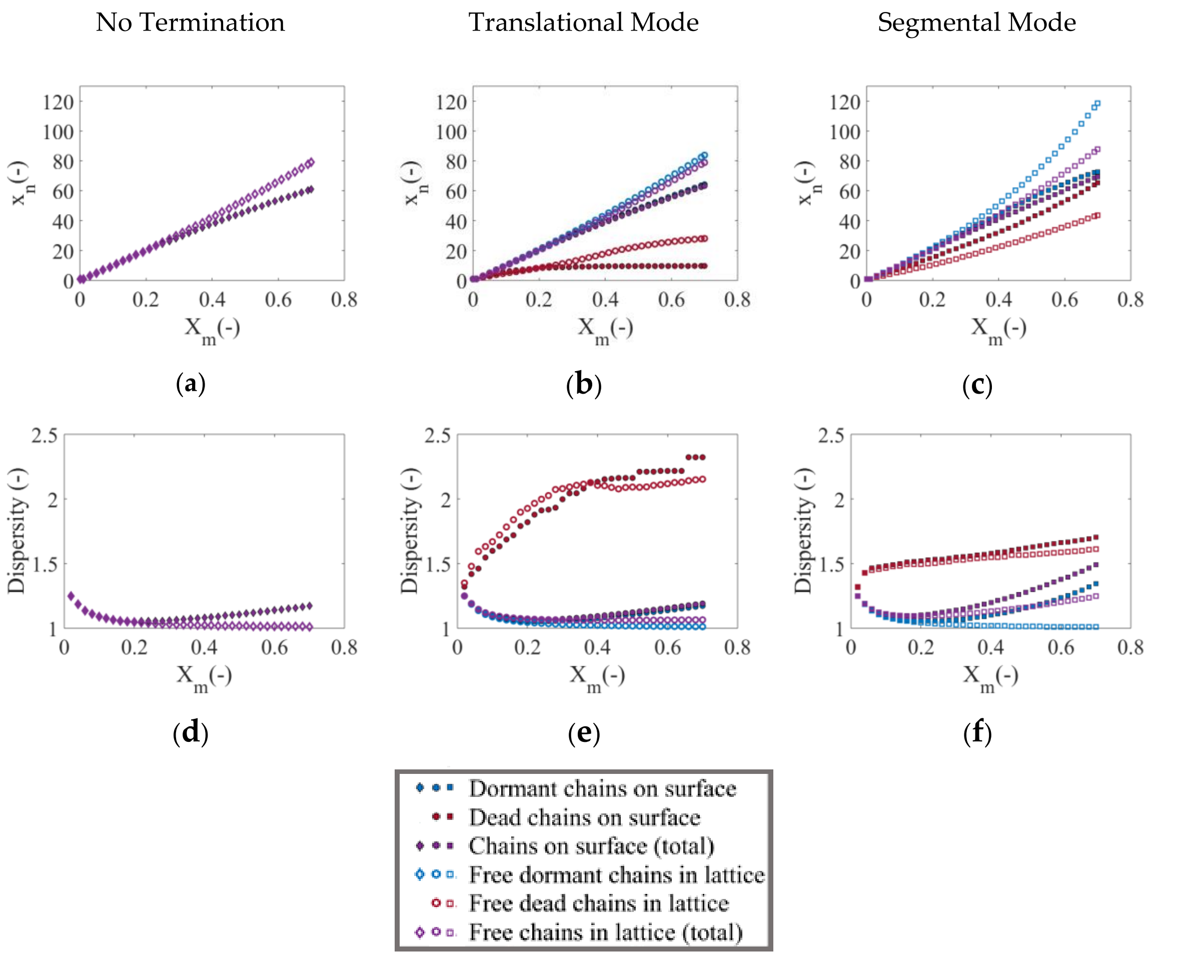


**Figure S8.** Study of the influence of the termination mode on the molecular properties of the polymerization products as a function of monomer conversion ($X_m$). Number average chain length ($x_n$; top figures) and dispersity (bottom figures) : (a) and (d) Absence of termination (diamond); (b) and (e) Translational mode (circles); (c) and (f) segmental mode (squares). Free polymer chains are represented as contoured empty symbols while surface-tethered polymer chains are represented as solid symbols. Total population (purple), dormant chains (blue) and dead chains (red). Same reference conditions as in **Figure S7**.

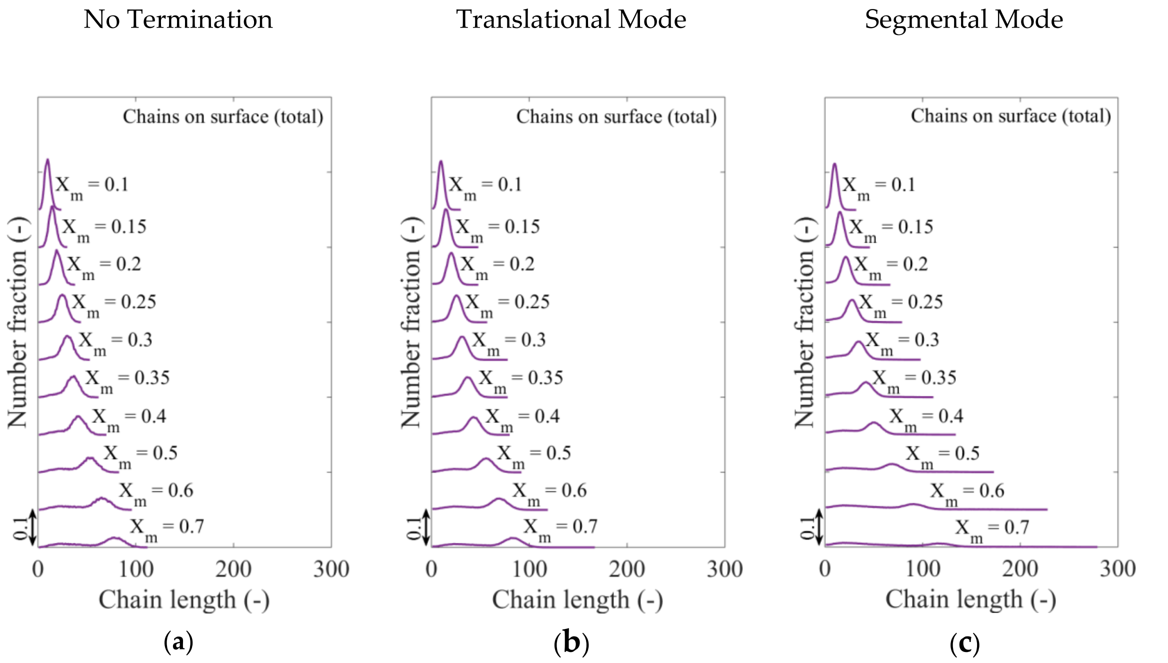


**Figure S9.** Number average chain length distributions of the total population of surface-tethered polymer chains in the SI-RDRP process as a function of monomer conversion. (a) Reference case of absence of termination reactions; (b) translational mode; (c) segmental mode. Reference conditions as in **Figure S7**.

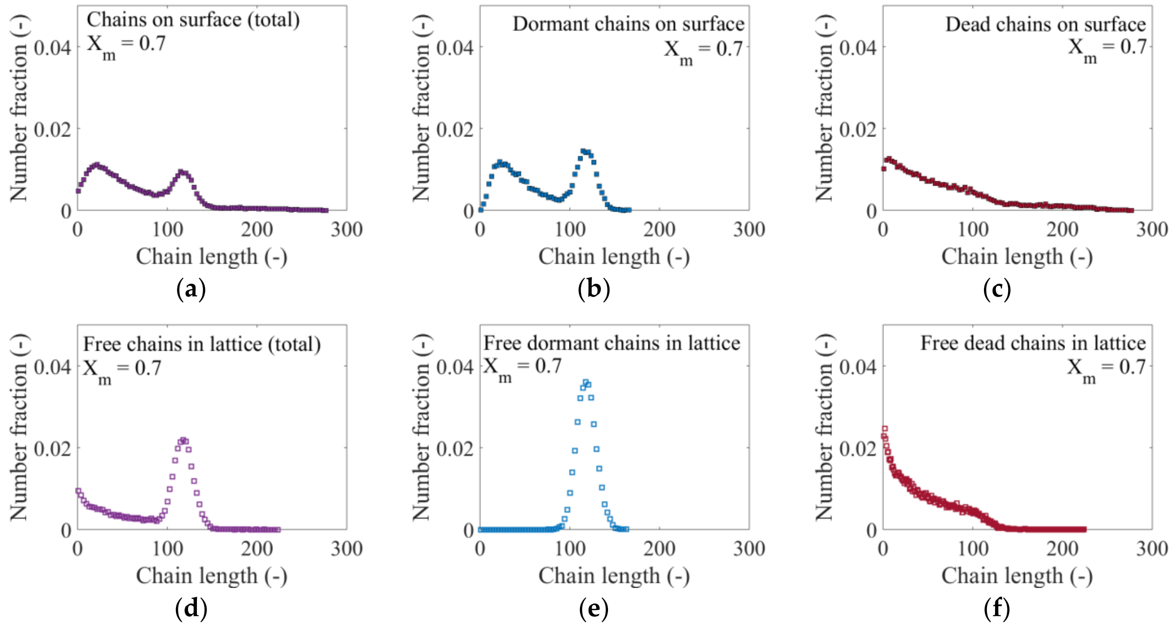


**Figure S10.** Top: Number chain length distribution of (a) all surface-tethered polymer chains (black-contoured full purple circles); (b) surface-tethered dormant chains (black-contoured full blue circles) and (c) surface-tethered dead polymer chains (black-contoured full red circles). Bottom: Number chain length distribution of (d) all free polymer chains (purple-contoured empty circles) (e) free dormant chains (blue-contoured empty circles) and (f) free dead polymer chains (red-contoured empty circles). In (a) and (d) the regions circled in red represent the contributions of dead polymer chains to the CLD of the total polymer species in both phases. These distributions are obtained at a monomer conversion $X_m$ = 0.7. Same reference conditions (**segmental mode**) conditions as in **Figure S7**.

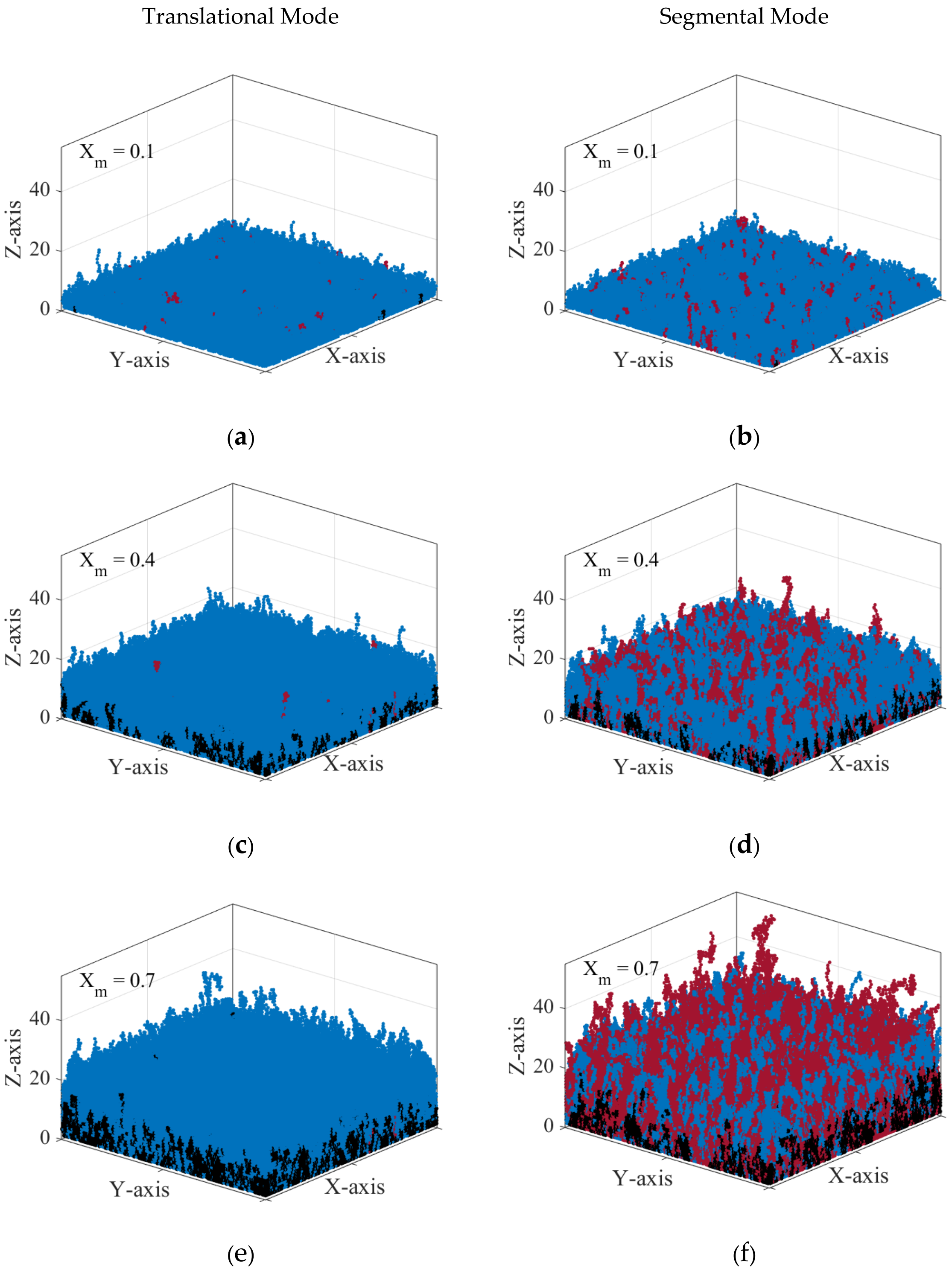


**Figure S11.** Snapshot of the polymer layer grown for two termination modes. The snapshots are taken a three different monomer conversion: (a) and (b) $X_m$ = 0.1; (c) and (d) $X_m$ = 0.4; (e) and (f) $X_m$ = 0.7. Surface-tethered dormant chains are represented in blue, dead chains on the surface are represented in red while the hindered dormant chains that cannot continue to propagate by the RDPR mechanism due to shielding are represented in black. Left column: translational mode; right column: segmental mode. Same reference conditions as in **Figure S8 and S9.**

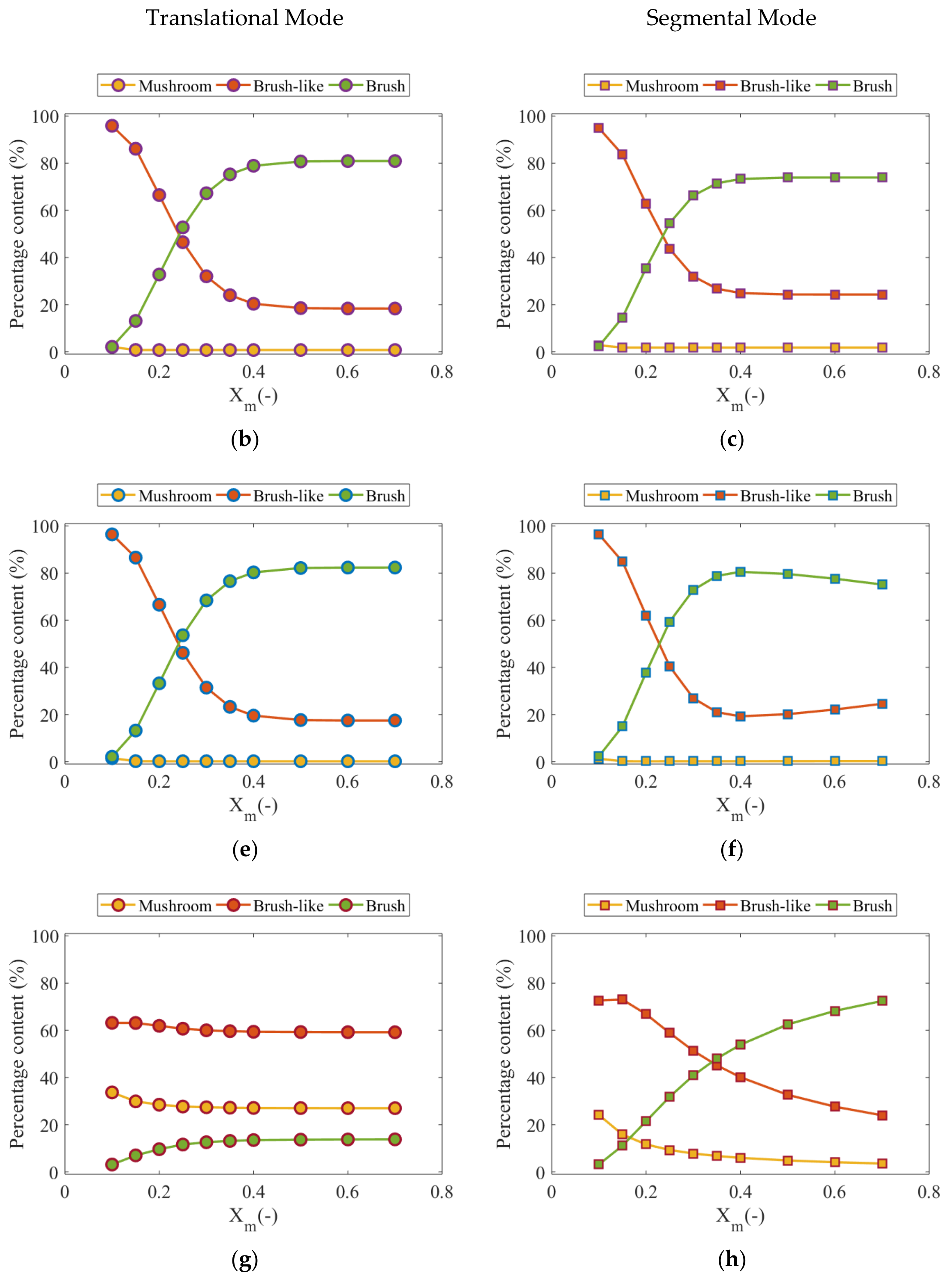


**Figure S12.** Variation of the percentage of the surface-tethered polymer chains as a function of monomer conversion ($X_m$) with mushroom (honey yellow), brush-like (orange) or brush (green) conformation for the two termination modes under consideration. Left column: translational mode (circles); Right column: segmental mode (squares). Color of the outline of the different symbols used: total population (purple), dormant chains (blue) and dead chains (red). Same reference conditions as in **Figure S8 – S10**.

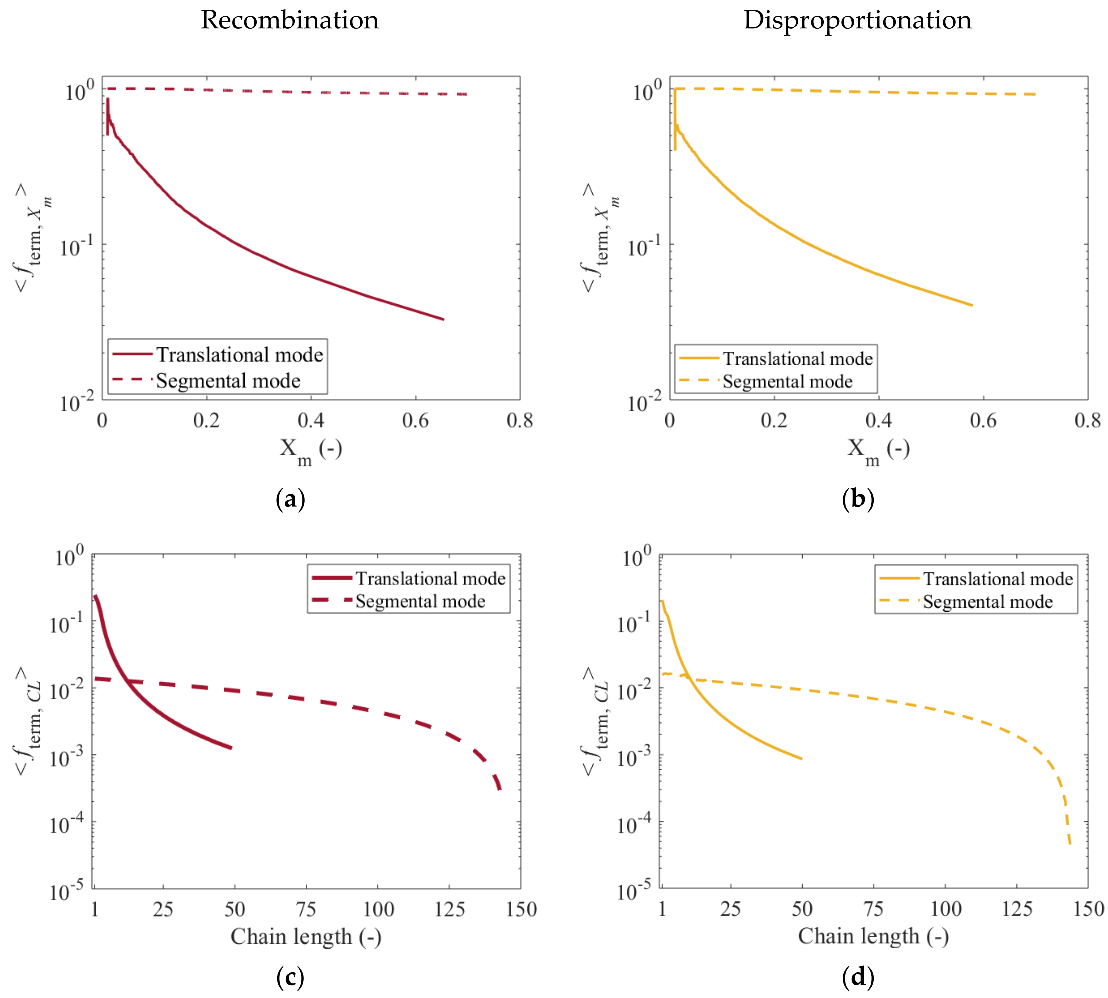


**Figure S13.** Variation of the fraction of successful termination reaction events averaged for all the polymer chains $<f_{term,X_m}>$ as a function of monomer conversion: (a) termination by recombination (red); (b) termination by disproportionation (honey yellow). Variation of the fraction of successful termination reaction events averaged for all monomer conversion $<f_{term,CL}>$ as a function of chain length: (c) termination by recombination (red); (d) termination by disproportionation (honey yellow). For all cases, a comparison of the two termination modes under consideration has been made. Same reference conditions as in **Figure S8 – S11**.

## References


1. Shaffer, J. S. Effects of Chain Topology on Polymer Dynamics - Bulk Melts. *J. Chem. Phys.* **1994,** 101 (5), 4205-4213 DOI: Doi 10.1063/1.467470.
2. Carmesin, I.; Kremer, K. The Bond Fluctuation Method - a New Effective Algorithm for the Dynamics of Polymers in All Spatial Dimensions. *Macromolecules* **1988,** 21 (9), 2819-2823 DOI: DOI 10.1021/ma00187a030.
